# Comparative efficacy of Chinese patent medicines in patients with carotid atherosclerotic plaque: a Bayesian network meta− analysis

**DOI:** 10.1186/s13020-023-00850-5

**Published:** 2023-11-20

**Authors:** Wenquan Su, Xiaolong Xie, Jiping Zhao, Qinhua Fan, Naijia Dong, Qingxiao Li, Yawei Du, Shengxian Wu

**Affiliations:** Dongzhimen Hospital, Beijing University of Chinese Medicine, Beijing, 100700 China

**Keywords:** Traditional Chinese medicine, Chinese patent medicines, Carotid atherosclerotic plaque, Network meta-analysis, Randomized controlled trials

## Abstract

**Background:**

Traditional Chinese patent medicines (TCPMs) have been widely used to treat carotid atherosclerotic plaque (CAP) in China. However, systematic evaluation of the clinical efficacy of TCPMs for CAP is still unknown, and the comparative efficacy of different TCPMs is unclear.

**Objectives:**

This study aims to compare and rank the effectiveness and safety of different TCPMs in treating CAP using a Bayesian network meta− analysis (NMA).

**Methods:**

This NMA was performed according to the Preferred Reporting Items for Systematic Reviews and Meta− Analyses (PRISMA) Extension Statement. Eight databases were searched from their inception to August 2023 for randomized controlled trials (RCTs). The articles regarding eligibility and extracted data were screened independently by two authors. The Cochrane Risk of Bias tool was used to evaluate quality and bias. The change of carotid artery intimal− medial thickness (IMT), carotid maximal plaque area, carotid atherosclerotic plaque Course score, serum lipid levels, CRP, and adverse events rate (AER) were used as outcomes. Data from each RCTs were first pooled using random− effect pairwise meta− analyses and illustrated as odds ratios (ORs) or standardized mean differences (SMDs) with 95% confidence interval (CI). NMAs were performed using Stata17.0 software and the GeMTC package of R software to evaluate the comparative effectiveness of TCPMs, and displayed as ORs or SMDs with 95% CI. A Bayesian hierarchical random− effects model was used to conduct NMAs using the Markov Chain Monte Carlo algorithm. The GRADE partially contextualised framework was applied for NMA result interpretation.

**Results:**

NMA included 27 RCT trials with 4131 patients and nine types of TCPMs. Pairwise meta− analyses indicated that Conventional Western medicine (CWM) + TCPM was superior to CWM in reducing the IMT (SMD: − 1.26; 95% CI − 1.59 to − 0.93), the carotid maximal plaque area (SMD − 1.27; 95% CI − 1.71, − 0.82) and the carotid atherosclerotic plaque Course score (SMD − 0.72; 95% CI 95% CI − 1.20, − 0.25). NMAs demonstrated that CWM + Jiangzhiling pill (JZL) with SUCRA 70.6% exhibited the highest effective intervention for reducing IMT. CWM + SXBX (Shexiang baoxin pill) was superior to other TCPMs in reducing the carotid maximal plaque area (83.0%), the atherosclerotic plaque Course score (92.5%), TC (95.6%) and LDL (92.6%) levels. CWM + NXT (Naoxintong capsule), CWM + XS (Xiaoshuang granules/enteric capsule), and CWM + ZBT (Zhibitai) were superior to other CPMs in improving TG (90.1%), HDL (86.1%), and CRP (92.6%), respectively. No serious adverse events were reported.

**Conclusions:**

For CAP patients, CWM + XSBX was among the most effective in reducing carotid maximal plaque area, atherosclerotic plaque Course score, TC and LDL levels, and CWM + JZL was the most effective in reducing IMT. Overall, CWM + XSBX may be considered an effective intervention for the treatment of CAP. This study provides reference and evidence for the clinical optimization of TCPM selection in CAP treatment. More adequately powered, well− designed clinical trials to increase the quality of the available evidence are still needed in the future due to several limitations.

**Supplementary Information:**

The online version contains supplementary material available at 10.1186/s13020-023-00850-5.

## Introduction

Carotid atherosclerotic plaque (CAP) is an important cause of carotid artery stenosis and has a high global prevalence. CAP global prevalence was approximately 21.1% in 2020, equivalent to 815.76 million people, and carotid artery stenosis global prevalence was approximately 1.5%, equivalent to 57.79 million people between the ages of 30 and 79 [1]. CAP prevalence between the ages of 30 and 79 was approximately 20.15%, equivalent to 199.83 million people in China [2]. The global burden of CAP is expected to increase as populations age, placing a huge burden on health care. Some guidelines have recommended CAP as a potentially useful predictor of coronary events and stroke [3]. CAP is an independent risk factor for stroke, and 45–50% of ischemic strokes are associated with bilateral CAP [4]. CAP is also detected in up to 80% of ischemic stroke patients [1]. According to a study, every 10% increase in plaque burden leads to a 2.26− fold higher risk of stroke recurrence (95% CI 1.03–4.96) [5]. Additionally, CAP is an effective predictor for coronary event incidence. A study involving 89 papers with 2,783 patients exhibited that CAP outperforms intimal− medial thickness (IMT) in predicting coronary artery disease, with a summary sensitivity of 80% and a summary specificity of 67%, regardless of the diagnostic technique [6]. CAP has become an important global public health concern, increasing the risk of cardiovascular and cerebrovascular disease. CAP increases as the global population ages and is highest among the elderly, significantly increasing the health care burden. However, several studies have discovered that CAP formation can be slowed, stopped, reversed, or even disappear, which has significant implications for improving human health and relieving the medical burden [7].

Currently, carotid endarterectomy (CEA), carotid stent placement (CAS), and optimal drug therapy (OMT) are the primary treatments for CAP and carotid artery stenosis [8]. Although surgical methods may improve stenosis caused by excessive CAP growth, these invasive treatments always carry surgical risks and complications, such as cervical hematoma, craniofacial nerve injury, cardiovascular events, cerebral hyperperfusion syndrome, and infection, and should be reserved for patients with significant syndromes, high stenosis, or vulnerable plaque. Additionally, a study has demonstrated that CEA reduced the risk of bilateral stroke by only 4.1% at five years compared to OMT [9]. Therefore, OMT, as a non− invasive treatment for CAP, is receiving increasing attention [10]. Statin is the central drug in OMT to stabilize and reverse atherosclerotic plaque. A three− dimensional ultrasound study to evaluate CAP has demonstrated regression of 90.25 ± 85.12 mm^3^ in CAP volume after three months of atorvastatin treatment, compared to a progression of 16.81 ± 74.10 mm^3^ on placebo (P < 0.0001) [11]. The effect of statin on reversing CAP progression depends on lowering the low− density lipoprotein cholesterol (LDL− C) levels. Expert consensus has recommended long− term intensive statin therapy to reduce LDL− C to 1.8 mmol/L and significantly increase HDL− C, potentially reversing atherosclerotic plaque, but inducing a 12% increased risk of new diabetes, a 5% increased risk of muscle disease and a two—to three− fold increased risk of severe liver damage [12]. An MRI assessment study revealed that statin therapy did not consistently reduce the CAP lipid content. The effect occurred primarily between years one and two, with little further reduction in year three [13]. Long− term intensive statin therapy carries a greater risk, especially for patients who use statins cautiously, such as the elderly, those with low body mass, abnormal liver and kidney function, and those with a history of adverse drug reactions. Therefore, there is an urgent need for complementary and alternative drugs to improve drug regimens of OMT further because the efficacy of statins in reversing CAP is not entirely satisfactory.

Traditional Chinese patent medicines (TCPMs) with reliable pharmaceutical ingredients and manufacturing processes have been widely used to treat chronic diseases as an important part of Traditional Chinese medicine (TCM) in China [14]. In 2018, a meta− analysis of 12 randomized controlled trials (RCT) articles, including 1,052 CAP patients, demonstrated that combined TCM and Western medicine are superior to Western medicine alone for treating CAP regarding clinical efficacy (OR = 3.07 [1.96, 4.81], *P* < 0.00001), IMT (OR =—0.09 [become an important global public health concern 0.10, − 0.08], *P* < 0.00001), course score (OR = − 0.96 [− 1.09, − 0.83], *P* < 0.00001), and plaque area (OR = − 0.20 [− 0.23, − 0.17], *P* < 0.00001)[15]. Guidelines have recommended that TCPMs combined with conventional Western medicine (CWM) to treat atherosclerotic disease, including coronary arteries, carotid and cerebral arteries [16, 17]. Among them, Tongxinluo capsule (TXL), Xiaoshuang granules/enteric capsule (XS), Naoxintong capsule (NXT), Xuesaitong capsule/soft capsule (XST), Jiangzhiling pill (JZL), Pushen capsule (PS), Shexiang baoxin pill (SXBX), Zhibitai (ZBT), and Dengzhan shengmai capsule (DZSM) were approved by the State Food and Drug Administration of China to treat symptoms of cerebrovascular disease, including dizziness, headache, stroke, aphasia, paralysis and fainting. In the treatment of CPA, TCPMs have the functions of tonifying qi, activating blood, resolving stasis, freeing the collateral vessels, resolving phlegm and resolving turbidity. According to Pharmacopoeia of the people’s Republic of China 2020, Table 1 presents the details of traditional effects of the included TCPMs. A vast number of randomized controlled trials have reported and published TCPMs for treating CAP [18, 19]. However, systematic evaluation of the clinical efficacy of TCPMs for CAP is still unknown, and the comparative effectiveness of different TCPMs is unclear. This study utilizes Bayesian network meta− analysis (NMA) to compare and rank different TCPMs to provide reference and evidence support for the clinical optimization of TCPM selection in CAP treatment.

**Table 1:** Ingredients and traditional effects of the included TCPMs

TCPMs	Ingredients (pin yin)	Traditional effects
Tongxinluo capsule (TXL)	*Panax ginseng* C.A.Mey. (Renshen), Hirudo (Shuizhi), Scorpio (Quanxie), *Paeonia lactiflora* Pall. (Chishao), Cicadae Periostracum (Chantui), Eupolyphaga Steleophaga (Tubie Chong), Scolopendra (Wugong), *Santalum album* L. (Tanxiang), *Dalbergia odorifera* T.C.Chen (Jiangxiang), *Boswellia ameero* Balf.f. (Ruxiang), *Ziziphus jujuba* Mill. (Suanzao Ren), *Cinnamomum camphora* (L.) J.Presl (Bingpian)	Tonifying qi, activating blood, freeing the collateral vessels to relieve pain
Xiaoshuang granules/enteric capsule (XS)	*Astragalus membranaceus* (Fisch.) Bunge (Huangqi), *Angelica sinensis* (Oliv.) Diels (Danggui), *Paeonia lactiflora* Pall. (Chishao), Pheretima (Dilong), *Ligusticum chuanxiong* S.H.Qiu, Y.Q.Zeng, K.Y.Pan, Y.C.Tang & J.M.Xu (Chuanxiong), *Prunus persica* (L.) Batsch (Taoren), *Carthamus tinctorius* L. (Honghua)	Tonifying qi, activating blood, freeing the collateral vessels
Naoxintong capsule (NXT)	*Astragalus membranaceus* (Fisch.) Bunge (Huangqi), *Paeonia lactiflora* Pall. (Chishao), *Salvia miltiorrhiza* Bunge (Danshen), *Angelica sinensis* (Oliv.) Diels (Danggui), *Ligusticum chuanxiong* S.H.Qiu, Y.Q.Zeng, K.Y.Pan, Y.C.Tang & J.M.Xu (Chuanxiong), *Prunus persica* (L.) Batsch (Taoren), *Carthamus tinctorius* L. (Honghua)､*Boswellia ameero* Balf.f. (Ruxiang), *Commiphora myrrha* (Nees) Engl. (Moyao), *Spatholobus suberectus* Dunn (Jixue Teng)､*Achyranthes bidentata* Blume (Niuxi)､*Cinnamomum cassia* (L.) J.Presl (Guizhi)､*Morus alba* L. (Sangzhi)､Pheretima (Dilong)､Scorpio (Quanxie)､Hirudo (Shuizhi)	Tonifying qi, activating blood, resolving stasis, freeing the collateral vessels
Xuesaitong capsule/soft capsule (XST)	Notoginseng Total Saponins (Sanqi Zongzaogan)	Activating blood, resolving stasis, activating collaterals
Jiangzhiling pill (JZL)	*Polygonum abbreviatum* Kom. (Heshouwu)､*Lycium barbarum* L. (Gouqizi), *Polygonatum kingianum* Collett & Hemsl. (Huangjing), *Crataegus pinnatifida* Bunge (Shanzha), *Cassia obtusifolia* L. (Juemingzi)	enriching the kidney, nourishing the liver, tonifying blood
Pushen capsule (PS)	*Polygonum abbreviatum* Kom. (Heshouwu), *Typha angustifolia* L. (Puhuang), *Salvia miltiorrhiza* Bunge (Danshen), *Ligusticum chuanxiong* S.H.Qiu, Y.Q.Zeng, K.Y.Pan, Y.C.Tang & J.M.Xu (Chuanxiong), *Paeonia lactiflora* Pall. (Chishao), *Crataegus pinnatifida* Bunge (Shanzha), *Alisma orientale* (Sam.) Juz. (Zexie)､*Codonopsis pilosula* (Franch.) Nannf. (Dangshen)	Activating blood, resolving stasis, enriching yin, resolving turbidity
Shexiang baoxin pill (SXBX)	Moschus (Rengong Shengxiang), Ginseng extract (Renshen Tiquwu), Bovis calculus artifactus (Rengong Niuhuang)､*Cinnamomum cassia* (L.) J.Presl (Rougui)､Liquidambar orientalis Mill. (Suhexiang)､Bufonis venenum (Chansu), *Cinnamomum camphora* (L.) J.Presl (Bingpian)	Opening the orifices with aroma, tonifying qi
Zhibitai (ZBT)	*Crataegus pinnatifida* Bunge (Shanzha)､*Alisma orientale* (Sam.) Juz. (Zexie)､*Atractylodes macrocephala* Koidz. (Baizhu)､Red rice (Hongqu)	Resolving phlegm, resolving stasis, fortifying the spleen, harmonizing the stomach
Dengzhan shengmai capsule (DZSM)	*Erigeron breviscapus* (Vaniot) Hand.− Mazz. (Xixin), *Panax ginseng* C.A.Mey.(Renshen),*Schisandra chinensis* (Turcz.) Baill. (Wuweizi), *Ophiopogon japonicus* (Thunb.) Ker Gawl. (Maidong)	Tonifying qi, enriching yin, activating blood

## Methods

### Protocol and registration

This NMA was performed per the Preferred Reporting Items for Systematic Reviews and Meta− Analyses (PRISMA) Extension Statement [20]. This study’s protocol was registered in the international prospective register of systematic reviews (PROSPERO) (CRD42022366012).

### Eligibility criteria

#### Study types

RCTs published in Chinese or English, regardless of blinding, publication status, were included.

#### Participant types

A patient was diagnosed with CAP, including hypertension, coronary atherosclerotic heart disease, and diabetes, using carotid ultrasound [21]. Age, gender, race, disease course, region, and nationality were unrestricted.

#### Intervention types

The experiment group was administrated TCPMs, regardless of dosage and treatment duration, combined with CWM per guidelines. Patients in the control group received CWM with or without a placebo (PBO) of TCPM or CWM plus another TCPM. Considering that patients with CAP were complicated with hyperlipidemia, hypertension, diabetes, coronary heart disease, cerebral infarction and other underlying diseases, the CWM was primarily used against antihypertensive, hypoglycemic, hypolipidemic, and anti− platelet aggregation.

#### Outcome types

The primary outcome was the change in indicators of carotid artery IMT at the end of treatment. The additional outcomes were the change in the carotid maximal plaque area, carotid atherosclerotic plaque Course score, serum levels of lipids, CRP, and adverse events rate (AER) at the end of treatment.

### Exclusion criteria

Studies that met the following criteria were excluded: (1) animal experiments, reviews, meta− analyses, retrospective studies, or case reports; (2) research data with serious errors or no access to the full text after seeking help online or contacting the corresponding author via email; (3) repeated publication (the first published article was retained); (4) studies with incomparable baseline data between the two groups; (5) studies with a high or unclear risk of bias in sequence generation according to the Cochrane Collaboration’s risk of bias tool; (6) interventions that were combined with other Chinese herbal medicines or common TCM technology, such as acupuncture, moxibustion, and massage; (7) several cases less than 60.

### Search strategy

We searched the following databases from their inception to August 2023. Chinese databases include CNKI, WanFang Data, VIP, and CBM, while English databases include PubMed, Embase, the Cochrane Library, and Web of Science. Additionally, other databases include clinical trial registries (WHO ICTRP, Clinical Trials, and ChiCTR) and Allied and Complementary Medicine Database (AMED). The literature search was constructed around search terms for “Chinese patent medicines”, “carotid atherosclerotic plaque”, and “randomized controlled trial” and adapted for each database as necessary. Additional file 1 provides a detailed and specific search strategy.

### Literature screening and data extraction

We screened the retrieved articles during the searches and two authors independently conducted a comprehensively assessment of potentially eligible articles according to the inclusion/exclusion criteria. The following data were extracted: author, year of publication, place of conduct, baseline characteristics (sex, age), sample size, intervention(s), comparison(s), course of treatment, and outcome(s). Any disagreement was resolved by discussion until a consensus was reached or by consulting a third author.

### Risk of bias assessment

All authors received advanced training and used the Cochrane Risk of Bias tool for quality assessment [22]. Each article was assessed independently by two authors. In case of disagreement between the two authors, a discussion was conducted or a third author was asked for advice. Seven items were used to assess biases covering six different domains for each included study. The bias domains and items were selection bias (random sequence generation and allocation concealment), performance bias (blinding of participants and personnel), detection bias (blinding of outcome assessment), attrition bias (incomplete outcome data), reporting bias (selective reporting), and other biases (other sources of bias). Each domain was assigned a risk of bias judgment within the included study using the labels 'low risk' of bias, 'high risk' of bias, or 'unclear' risk of bias.

### Statistical analysis

We conducted a head− to− head comparisons pairwise meta− analyses between CWM combined with TCPM and CWM using Review Manager 5.3. We conducted an NMA analysis using Stata17.0 software and the GeMTC package of R software, applying the Markov Chain Monte Carlo algorithm and a Bayesian hierarchical random− effects model [23]. The results were presented as odds ratios (ORs) with 95% confidence intervals (CIs) for dichotomous variables, and the standardized mean differences (SMDs) with 95% CIs for continuous variables. If the range of 95% CIs of ORs did not cross 1 and 95% CIs of SMDs did not cross 0, then the differences between the groups would be considered statistically significant. The model was used four chains and 50,000 iterations, with the initial 20,000 iterations discarded as the starting point for annealing to eliminate the influence of initial value [24]. Using the surface under the cumulative ranking curve (SUCRA), we sorted the probabilities of different interventions of each outcome [25]. We used the node− splitting analysis to separate mixed evidence into direct and indirect evidence, to evaluate the consistency of the model. We also conducted the multi− dimensional efficacy analysis integrate multiple outcomes, and obtain the optimal intervention. Furthermore, we used a comparison− adjusted funnel plot to detect the publication bias of included RCTs [26]. The interventions were stratified according to the certainty of evidence supporting their relative efficacy which was graded using the GRADE NMA rating system.

## Results

### Literature screening

**Fig. 1:**
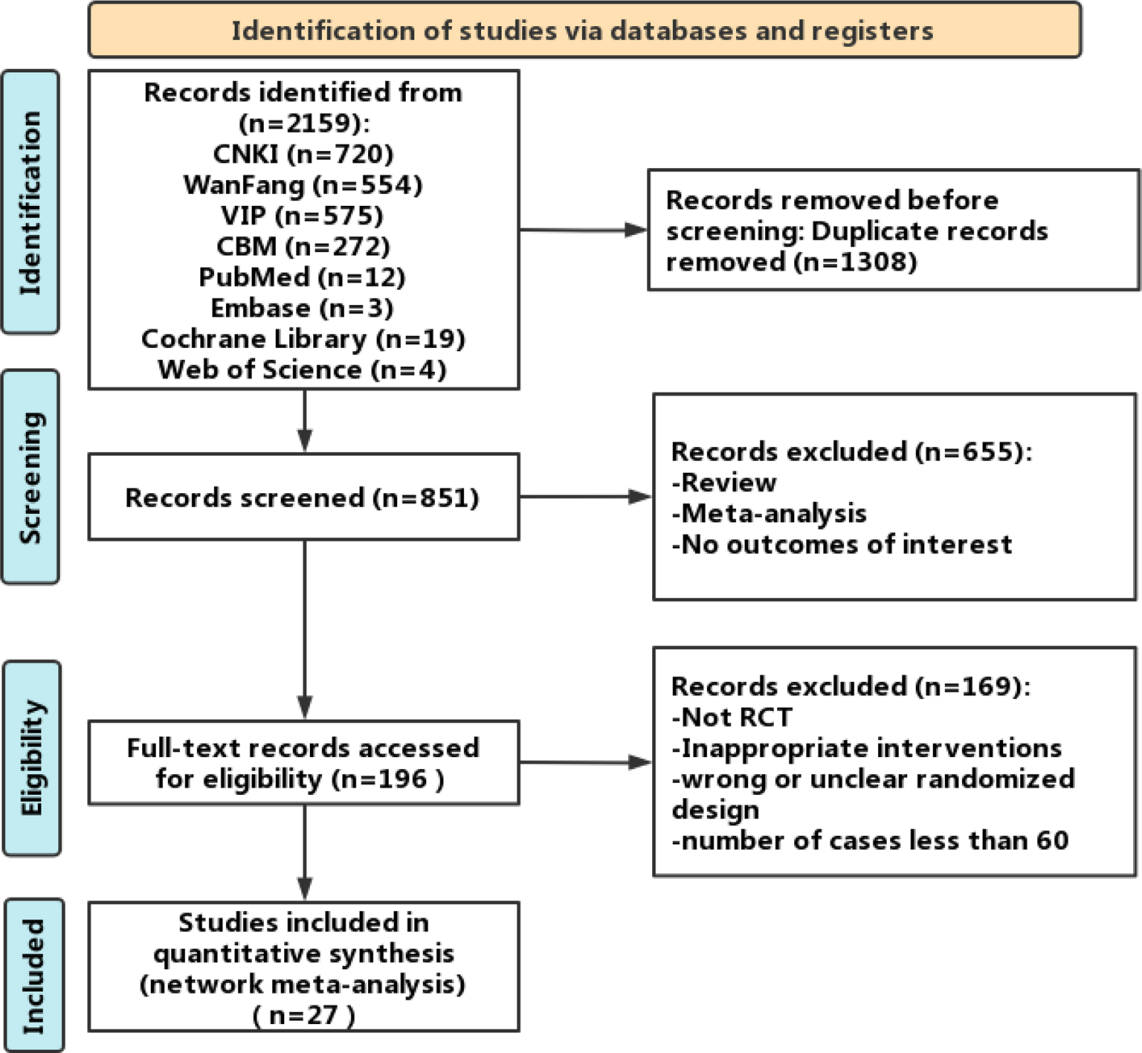
Flowchart of the literature screening process

Initially, the search strategy yielded 2,159 articles. Duplication resulted in the removal of 1,308 articles. The remaining 851 articles were filtered further and excluded according to the eligibility and exclusion criteria. After rereading the full texts, 27 studies remained for quantitative synthesis [27–53]. Figure 1 presents the details of the literature screening process.

### Study characteristics

There were 25 Chinese articles and two English articles involving 11 interventions. All the articles were conducted in China. Overall, 4,131 patients (2,069 in the experimental control group and 2,062 in the control groups). Nine kinds of CPMs were enrolled: Tongxinluo capsule (TXL), Xiaoshuang granules/enteric capsule (XS), Naoxintong capsule (NXT), Xuesaitong capsule/soft capsule (XST), Jiangzhiling pill (JZL), Pushen capsule (PS), Shexiang baoxin pill (SXBX), Zhibitai (ZBT), and Dengzhan shengmai capsule (DZSM). Table 1 presents the details of ingredients of the included TCPMs. Plant names have been checked with www.theplantlist.org.

Most articles were open− label trials except for two double− blind trials. Both groups were based on CWM, with TCPM addition in the treatment group and PBO addition or blank to the control group, including CWM + TXL vs. CWM + PBO (n = 1), CWM + TXL vs. CWM (n = 6), CWM + XS vs. CWM (n = 2), CWM + NXT vs. CWM (n = 3), CWM + XST vs. CWM (n = 2), CWM + JZL vs. CWM (n = 2), CWM + PS vs. CWM (n = 3), CWM + SXBX vs. CWM (n = 3), CWM + ZBT vs. CWM (n = 3), and CWM + DZSM vs. CWM (n = 2). There were no significant differences in gender and age between the study groups with comparable baselines, and most were middle− aged or elderly. Table 2 presents the details of the included study characteristics.

**Table 2:** Characteristics of the studies included in this network meta− analysis

Study ID	Study design	Sample size (T/C)	Sex (M/F)	Average age	Inventions	Course	Dosage	Outcomes
[52]	RCT	1212 (607/605)	T: 367/240 C: 355/250	T: 61.4 ± 8.4 C: 61.4 ± 8.2	T: CWM + TXL C: CWM + PBO	24 months	1560 mg bid po	1,2,4,5,6,7,8,9
[40]	RCT	168 (84/84)	T: 56/28 C:55/29	T: 58.6 ± 3.2 C: 59.1 ± 2.7	T: CWM + TXL C: CWM	6 months	1040 mg tid po	1,2,8
[41]	RCT	64 (32/32)	T: 18/14 C:15/17	T: 57.4 ± 6.7 C: 56.8 ± 7.1	T: CWM + TXL C: CWM	6 months	1040 mg tid po	1,2,4,5,6,7,8,9
[28]	RCT	106 (53/53)	T: 33/20 C:31/22	T: 62.5 ± 9.8 C: 63.8 ± 9.4	T: CWM + TXL C: CWM	12 months	780 mg tid po	1,2,3,9
[35]	RCT	60 (30/30)	T: 17/13 C:20/10	T: 58.6 ± 8.3 C: 61.0 ± 7.6	T: CWM + TXL C: CWM	12 months	780 mg tid po	1,3,4,5,6,7,8,9
[30]	RCT	120 (60/60)	T: 33/27 C:29/31	T: 53.4 ± 12.8 C: 55.8 ± 11.7	T: CWM + TXL C: CWM	5 months	780 mg tid po	1,2,4,5,6,7,9
[45]	RCT	70 (35/35)	T: 17/18 C:19/16	T: 61.2 ± 11.5 C: 63.5 ± 10.7	T: CWM + TXL C: CWM	3 months	1040 mg tid po	1,4,5,6,7,8,9
[48]	RCT	90 (45/45)	–	–	T: CWM + XS C: CWM	3 months	400 mg tid po	1
[36]	RCT	192 (96/96)	T: 58/38 C:56/40	T: 62.1 ± 8.3 C: 61.9 ± 8.1	T: CWM + XS C: CWM	6 months	400 mg tid po	1,4,5,6,7,9
[46]	RCT	110 (55/55)	–	–	T: CWM + NXT C: CWM	6 months	1200 mg tid po	1
[39]	RCT	134 (67/67)	T: 38/29 C:35/32	T: 58.7 ± 12.4 C: 64.3 ± 13.5	T: CWM + NXT C: CWM	6 months	1200 mg tid po	1,4,5,6,7,8
[31]	RCT	80 (40/40)	–	–	T: CWM + NXT C: CWM	3 months	1600 mg tid po	1,2,8
[34]	RCT	71 (36/35)	T: 21/15 C:20/15	T: 64.8 ± 12.4 C: 64.3 ± 13.5	T: CWM + XST C: CWM	3 months	100 mg tid po	1,3,9
[38]	RCT	106 (53/53)	T: 30/23 C:31/22	T: 68.0 ± 4.1 C: 68.5 ± 4.3	T: CWM + XST C: CWM	6 months	100 mg tid po	1,2,4,5,6
[53]	RCT	100 (50/50)	T: 25/25 C:23/27	T: 56.0 ± 10.0 C: 55.0 ± 11.0	T: CWM + JZL C: CWM	3 months	8000 mg bid po	1,2,4,5,6,7,9
[51]	RCT	186 (94/92)	T: 54/40 C:52/40	T: 68.1 ± 1.4 C: 67.2 ± 1.1	T: CWM + JZL C: CWM	12 months	1000 mg tid po	1,2,4,5,6,7,9
[49]	RCT	145 (73/72)	T: 45/28 C:45/27	T: 61.1 ± 7.5 C: 61.1 ± 7.5	T: CWM + PS C: CWM	4 months	1000 mg tid po	1,3,4,5,6,7
[27]	RCT	76 (38/38)	T: 25/13 C:26/12	T: 64.1 ± 4.2 C: 63.3 ± 5.2	T: CWM + PS C: CWM	12 months	1000 mg tid po	1,2, 4,5,6,7,8,9
[37]	RCT	73 (37/36)	–	–	T: CWM + PS C: CWM	6 months	1000 mg tid po	1,2,4,5,6,7,8,9
[44]	RCT	80 (39/41)	T: 24/15 C:25/16	T: 74.2 ± 15.8 C: 72.7 ± 12.4	T: CWM + SXBX C: CWM	6 months	450 mg tid po	1,2,3,4,5,6,7,8,9
[32]	RCT	116 (58/58)	T: 32/26 C:33/25	T: 66.0 ± 8.2 C: 65.2 ± 8.0	T: CWM + SXBX C: CWM	3 months	450 mg tid po	1,2,4,5,6
[33]	RCT	62 (32/30)	T: 19/13 C:18/12	T: 59.0 ± 7.0 C: 58.0 ± 7.5	T: CWM + SXBX C: CWM	12 months	450 mg tid po	1,4,5,6,7,9
[47]	RCT	180 (90/90)	T: 50/40 C:52/38	T: 67.9 ± 4.3 C: 68.7 ± 3.7	T: CWM + ZBT C: CWM	6 months	240 mg bid po	1,3,4,5,6,7,9
[42]	RCT	124 (62/62)	T: 32/30 C:35/27	T: 62.3 ± 7.9 C: 61.6 ± 7.3	T: CWM + ZBT C: CWM	6 months	480 mg bid po	1,2,4,5,6,7,8,9
[50]	RCT	60 (30/30)	T: 17/13 C:15/15	T: 70.3 ± 9.3 C: 70.2 ± 10.2	T: CWM + ZBT C: CWM	3 months	240 mg bid po	1,2,3,4,5,6,7
[43]	RCT	150 (75/75)	T: 34/41 C:42/33	T: 64.4 ± 7.5 C: 64.7 ± 6.9	T: CWM + DZSM C: CWM	12 months	360 mg tid po	1,3,4,5,6,7,9
[29]	RCT	196 (98/98)	T: 47/51 C:50/48	T: 67.8 ± 5.3 C: 68.2 ± 5.4	T: CWM + DZSM C: CWM	0.5 months	360 mg tid po	1,2,4,5,6,7,9

*RCT* randomized controlled trial, *T* treatment group, *C* control group, *M* male, *F* female, *CWM* conventional western medicine, *PBO* placebo, *TXL* Tongxinluo capsule, *XS* Xiaoshuang granules/enteric capsule, *NXT* Naoxintong capsule, *XST* Xuesaitong capsule/soft capsule, *JZL* Jiangzhiling pill, *PS* Pushen capsule, *SXBX* Shexiang baoxin pill, *ZBT* Zhibitai, *DZSM* Dengzhan shengmai capsule. 1.carotid artery intimal− medial thickness (IMT), 2. carotid maximal plaque area, 3. carotid atherosclerotic plaque course score, 4. total cholesterol (TC), 5. Triglyceride (TG), 6. low density lipoprotein (LDL), 7. high density lipoprotein (HDL), 8. C− reactive protein (CRP), 9. adverse events rate (AER)

### Risk of bias assessment

**Fig. 2:**
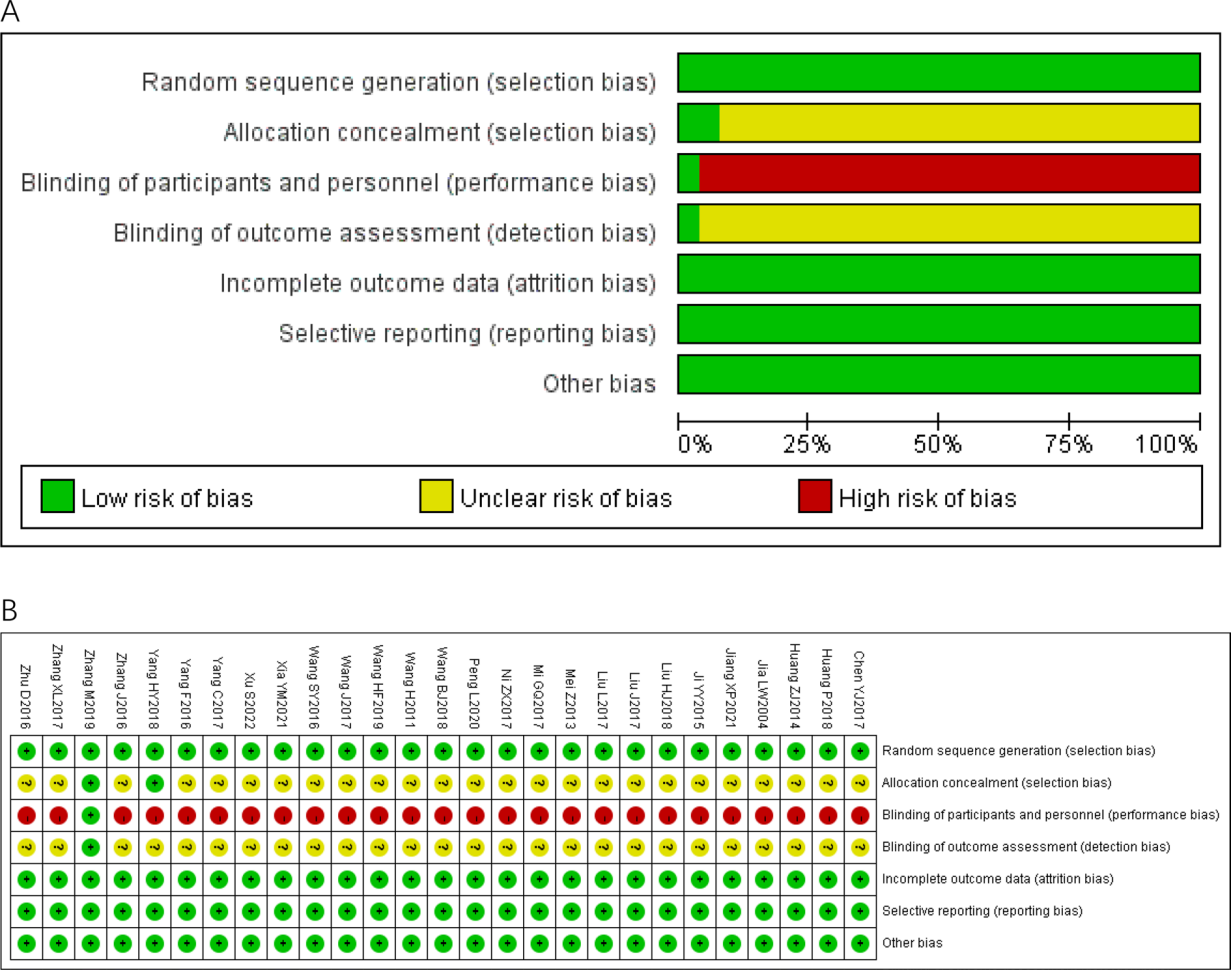
Risk of bias graph of the included RCT **A**: the risk of bias graph; **B**: the risk of bias summary

All the included trials reported ‘randomly allocating’ participants, generating random sequences using random number tables or computer− based or lottery methods, so they were evaluated as "low risk." Two trials reported allocation concealment, evaluated as "low risk," and the other studies did not mention allocation concealment and were evaluated as "uncertain risk." One trial reported double− blind trials were evaluated as "low risk," and the other studies did not mention blinding was evaluated as "high risk" or "uncertain risk". All trials had complete data, no selective reporting or other risk bias, and were all evaluated as "low risk." Fig. 2A depicts the risk bias assessment results. Figure 2B provides the detailed and specific risk of bias assessment.

### Outcomes

#### Pairwise meta-analysis

**Fig. 3:**
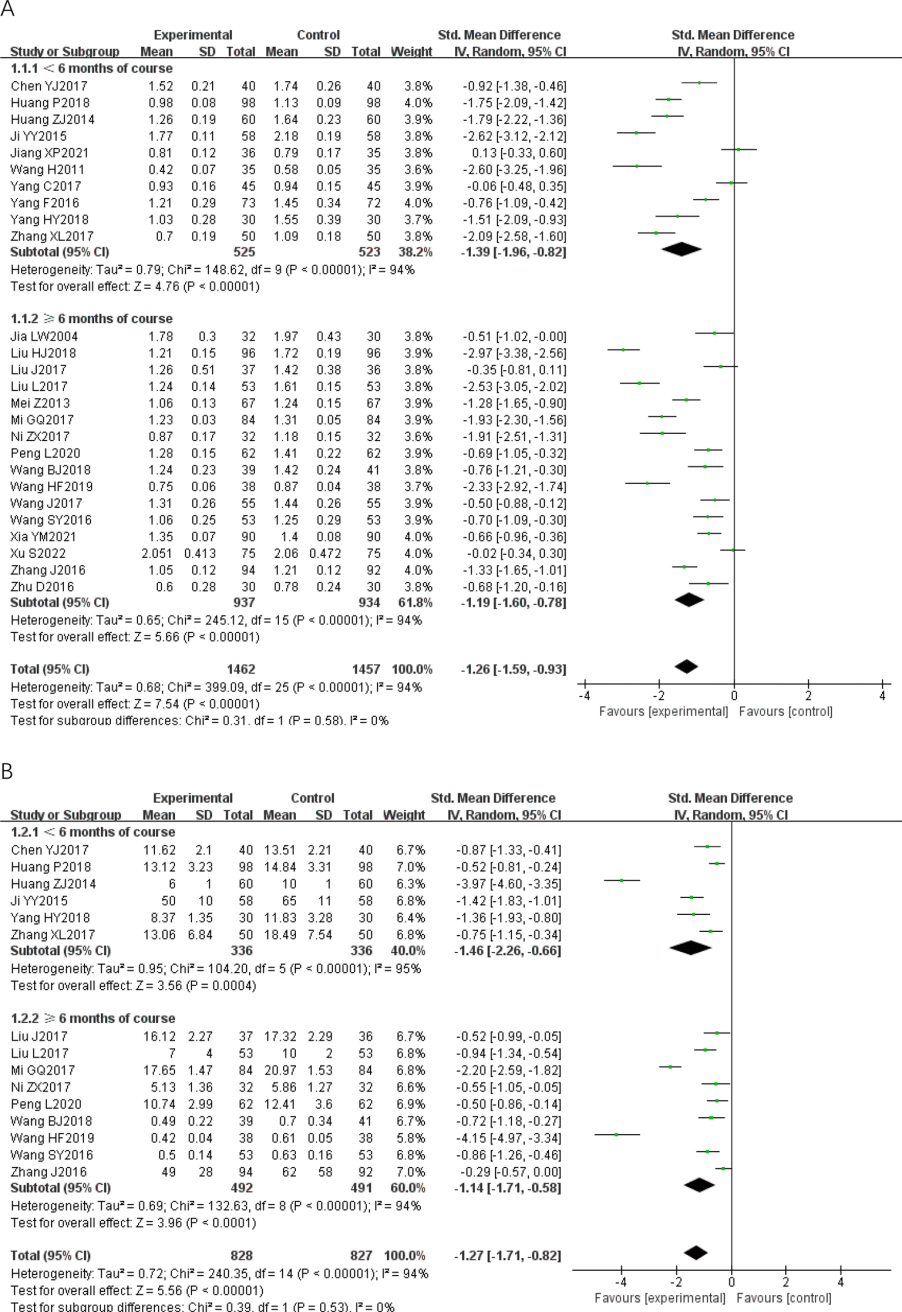

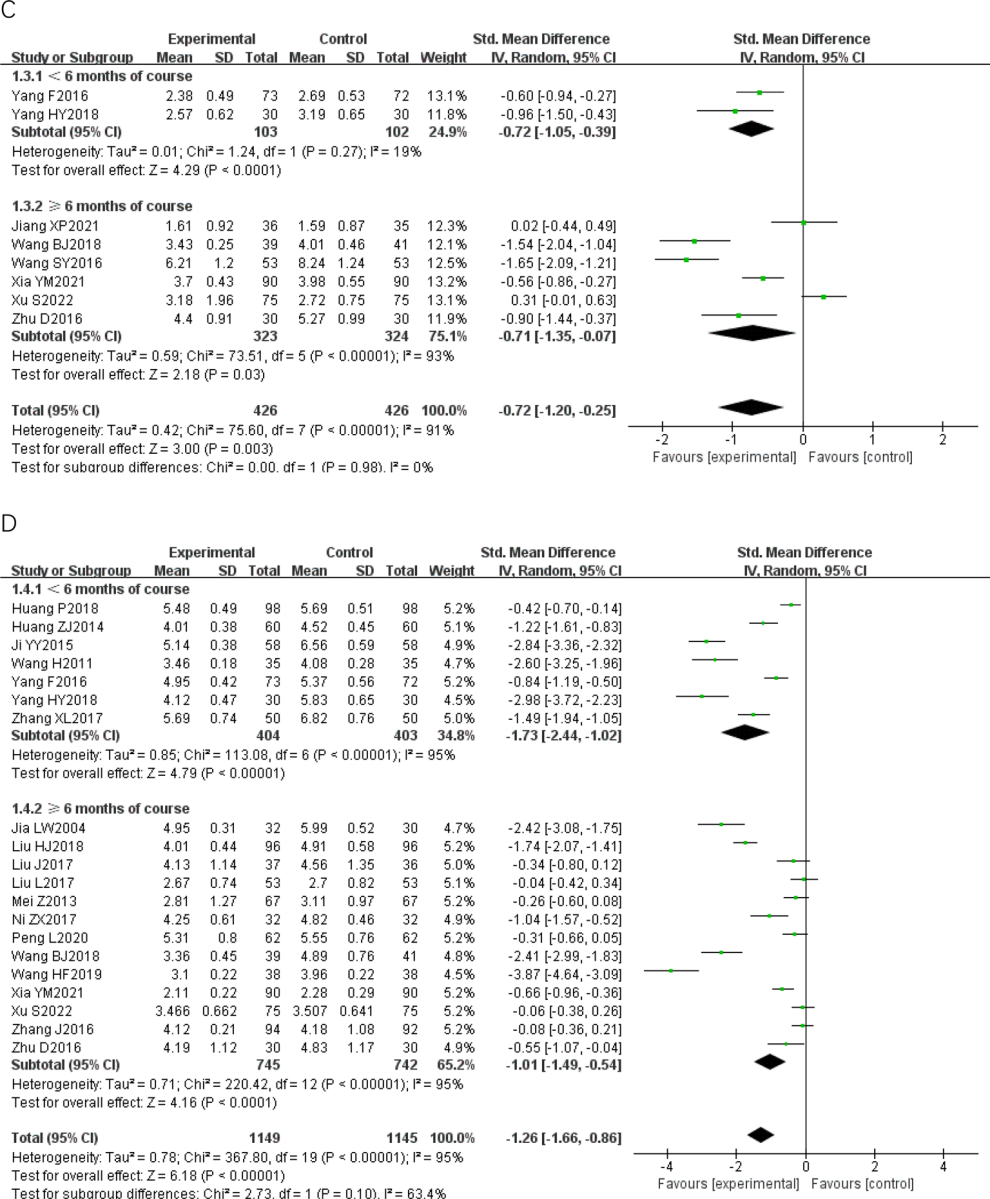

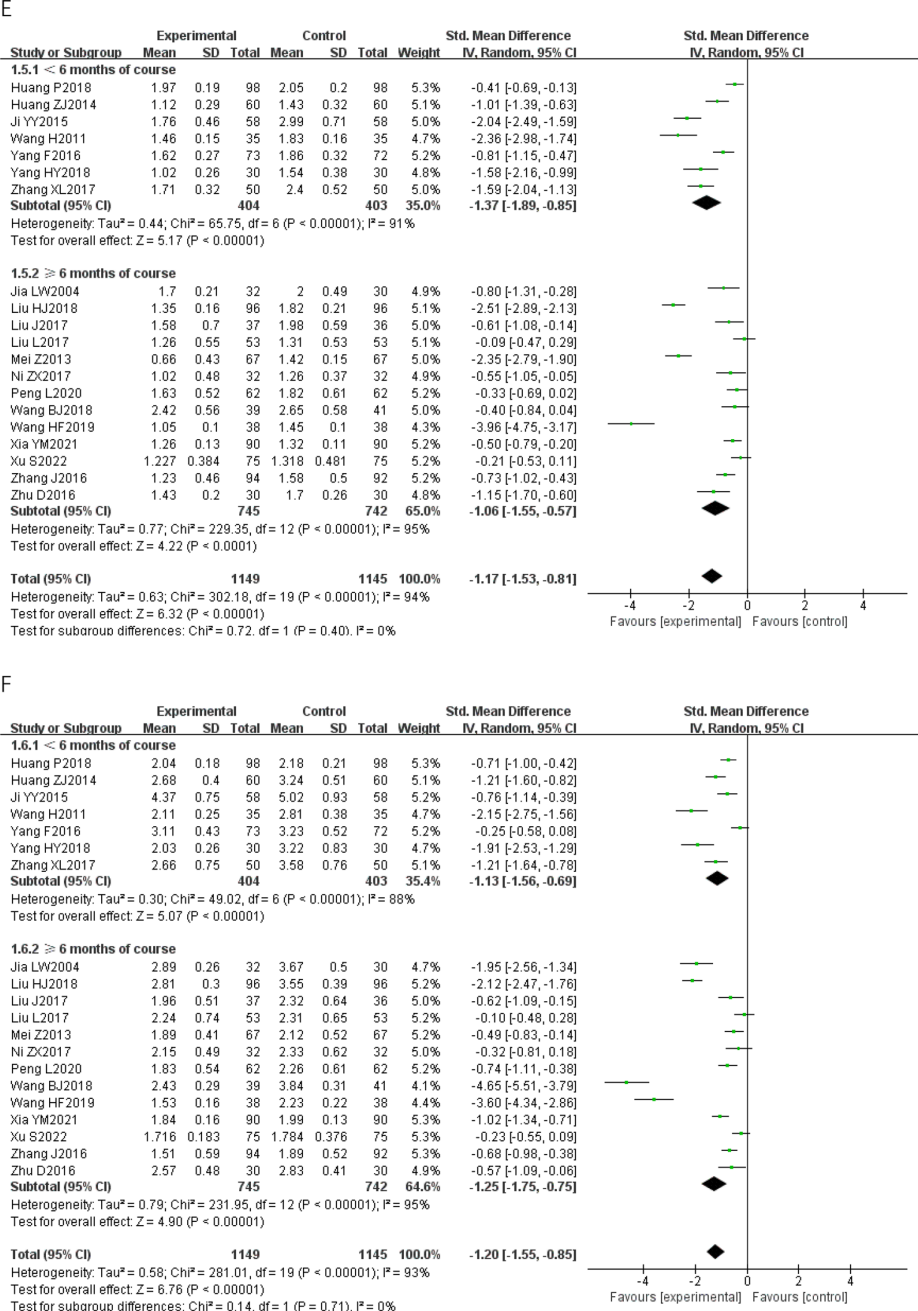

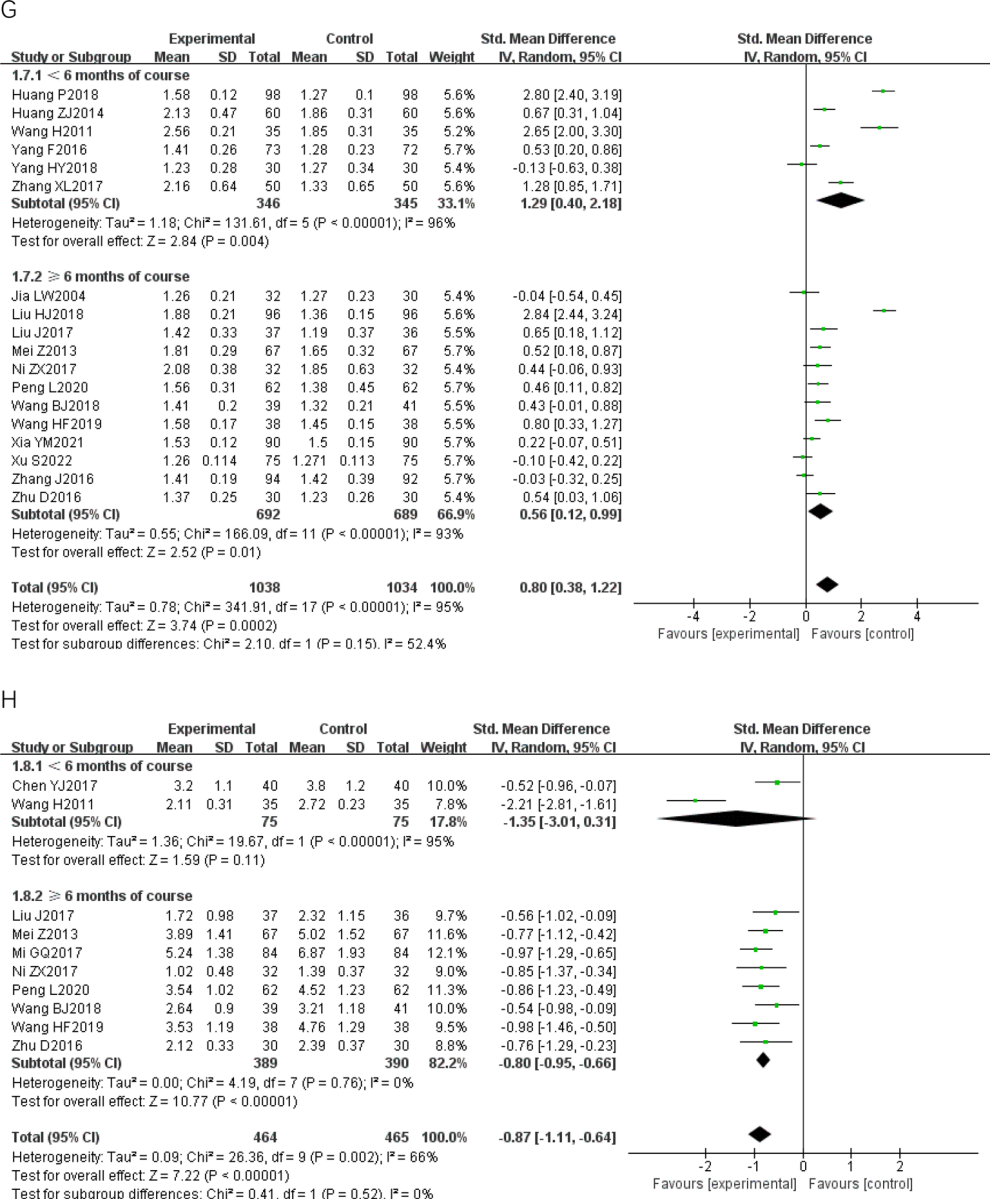
Forest plot of Pairwise meta-analysis. **A**: IMT; **B**: carotid maximal plaque area; **C**: carotid atherosclerotic plaque course score; **D**: TC; **E**: TG; **F**: LDL; **G**: HDL; **H**: CRP; *IMT* carotid artery intimal- medial thickness, *TC* total cholesterol, *TG* Triglyceride, *LDL* low density lipoprotein, *HDL* high density lipoprotein, *CRP* C− reactive protein, *AER* adverse events rate

We conducted eight pairwise meta− analyses comparing the effects of CWM and CWM combined with TCPM on improving the IMT, the carotid maximal plaque area, the carotid atherosclerotic plaque Course score, blood lipids, and CRP (Fig. 3). We assessed the certainty of the evidence for each outcome under the GRADE framework. The quality of the evidence for all of these comparisons was rated as low. The detailed GRADE assessment was presented in Table 3.

**Table 3:** GRADE assessment

Outcome	№ of studies	Certainty assessment						Effect		Certainty
		Study design	Risk of bias	Inconsistency	Indirectness	Imprecision	Publication bias	№ of individuals	Rate (95% CI)	
< 6 months of course										
IMT	10	RCT	Serious	Not serious	Not serious	Not serious	Strongly suspected	1048	SMD − 1.39 (− 1.96, − 0.82)	⨁⨁◯◯Low
Carotid maximal plaque area	6	RCT	Serious	Not serious	Not serious	Not serious	Strongly suspected	672	SMD − 1.46 (− 2.26, − 0.66)	⨁⨁◯◯Low
Carotid atherosclerotic plaque course score	2	RCT	Serious	Not serious	Not serious	Not serious	Strongly suspected	205	SMD − 0.72 (− 1.05, − 0.39)	⨁⨁◯◯Low
TC	7	RCT	Serious	Not serious	Not serious	Not serious	Strongly suspected	807	SMD − 1.73 (− 2.44, − 1.02)	⨁⨁◯◯Low
TG	7	RCT	Serious	Not serious	Not serious	Not serious	Strongly suspected	807	SMD − 1.37 (− 1.89, − 0.85)	⨁⨁◯◯Low
LDL	7	RCT	Serious	Not serious	Not serious	Not serious	Strongly suspected	807	SMD − 1.13 (− 1.56, − 0.69)	⨁⨁◯◯Low
HDL	6	RCT	Serious	Not serious	Not serious	Not serious	Strongly suspected	691	SMD 1.29 (0.4, 2.18)	⨁⨁◯◯Low
CRP	2	RCT	Serious	Not serious	Not serious	Not serious	Strongly suspected	150	SMD − 1.35 (− 3.01, 0.31)	⨁⨁◯◯Low
≥ 6 months of course										
IMT	16	RCT	Serious	Not serious	Not serious	Not serious	Strongly suspected	1871	SMD − 1.19 (− 1.6, − 0.87)	⨁⨁◯◯Low
Carotid maximal plaque area	9	RCT	Serious	Not serious	Not serious	Not serious	Strongly suspected	983	SMD − 1.14 (− 1.71, − 0.58)	⨁⨁◯◯Low
Carotid atherosclerotic plaque course score	6	RCT	Serious	Not serious	Not serious	Not serious	Strongly suspected	647	SMD − 0.71 (− 1.35, − 0.07)	⨁⨁◯◯Low
TC	13	RCT	Serious	Not serious	Not serious	Not serious	Strongly suspected	1487	SMD − 1.01 (− 1.49, − 0.54)	⨁⨁◯◯Low
TG	13	RCT	Serious	Not serious	Not serious	Not serious	Strongly suspected	1487	SMD − 1.06 (− 1.55, − 0.57)	⨁⨁◯◯Low
LDL	13	RCT	Serious	Not serious	Not serious	Not serious	Strongly suspected	1487	SMD − 1.25 (− 1.75, − 0.75)	⨁⨁◯◯Low
HDL	12	RCT	Serious	Not serious	Not serious	Not serious	Strongly suspected	1381	SMD 0.56 (0.12, 0.99)	⨁⨁◯◯Low
CRP	8	RCT	Serious	Not serious	Not serious	Not serious	Strongly suspected	779	SMD − 0.80 (− 0.95, − 0.66)	⨁⨁◯◯Low

*RCT* randomized controlled trial

Compared to CWM, CWM combined with TCPM had a stronger effect in reducing the IMT [26 RCTs; SMD − 1.26 (95% CI − 1.59, − 0.93); p < 0.00001; I^2 ^= 94%; low− quality of evidence] (Fig. 3A), decreasing the carotid maximal plaque area [15 RCTs; SMD − 1.27 (95% CI − 1.71, − 0.82); p < 0.00001; I^2 ^= 94%; low− quality of evidence] (Fig. 3B), lowering the carotid atherosclerotic plaque Course score [8 RCTs; SMD − 0.72 (95% CI − 1.20, − 0.25); p < 0.00001; I^2 ^= 91%; low− quality of evidence] (Fig. 3C), lowering the TC [20 RCTs; SMD − 1.26 (95% CI − 1.66, − 0.86); p < 0.00001; I^2 ^= 95%; low− quality of evidence] (Fig. 3D), lowering the TG [20 RCTs; SMD 1.17 (95% CI − 1.53, − 0.81); p < 0.00001; I^2 ^= 94%; low− quality of evidence] (Fig. 3E), lowering the LDL [20 RCTs; SMD − 1.20 (95% CI − 1.55, − 0.85); p < 0.00001; I^2 ^= 93%; low− quality of evidence] (Fig. 3F), raising the HDL [18 RCTs; SMD 0.80 (95% CI 0.38, 1.22); p < 0.00001; I^2 ^= 95%; low− quality of evidence] (Fig. 3G), and lowering the CRP [10 RCTs; SMD − 0.87 (95% CI − 1.11, − 0.64); p = 0.002; I^2 ^= 66%; low− quality of evidence] (Fig. 3H). Substantial heterogeneity was observed in all results.

We conducted sensitivity analysis comparing pooled results from “ < 6 months of course” and “ ≥ 6 months of course” is illustrated in Fig. 3. There was no significant subgroup difference between the two groups, implying that the difference in length of course did not influence the pooled results on improving the IMT, the carotid maximal plaque area, the carotid atherosclerotic plaque Course score, blood lipids, and CRP.

### Network meta− analysis

#### IMT

**Fig. 4:**
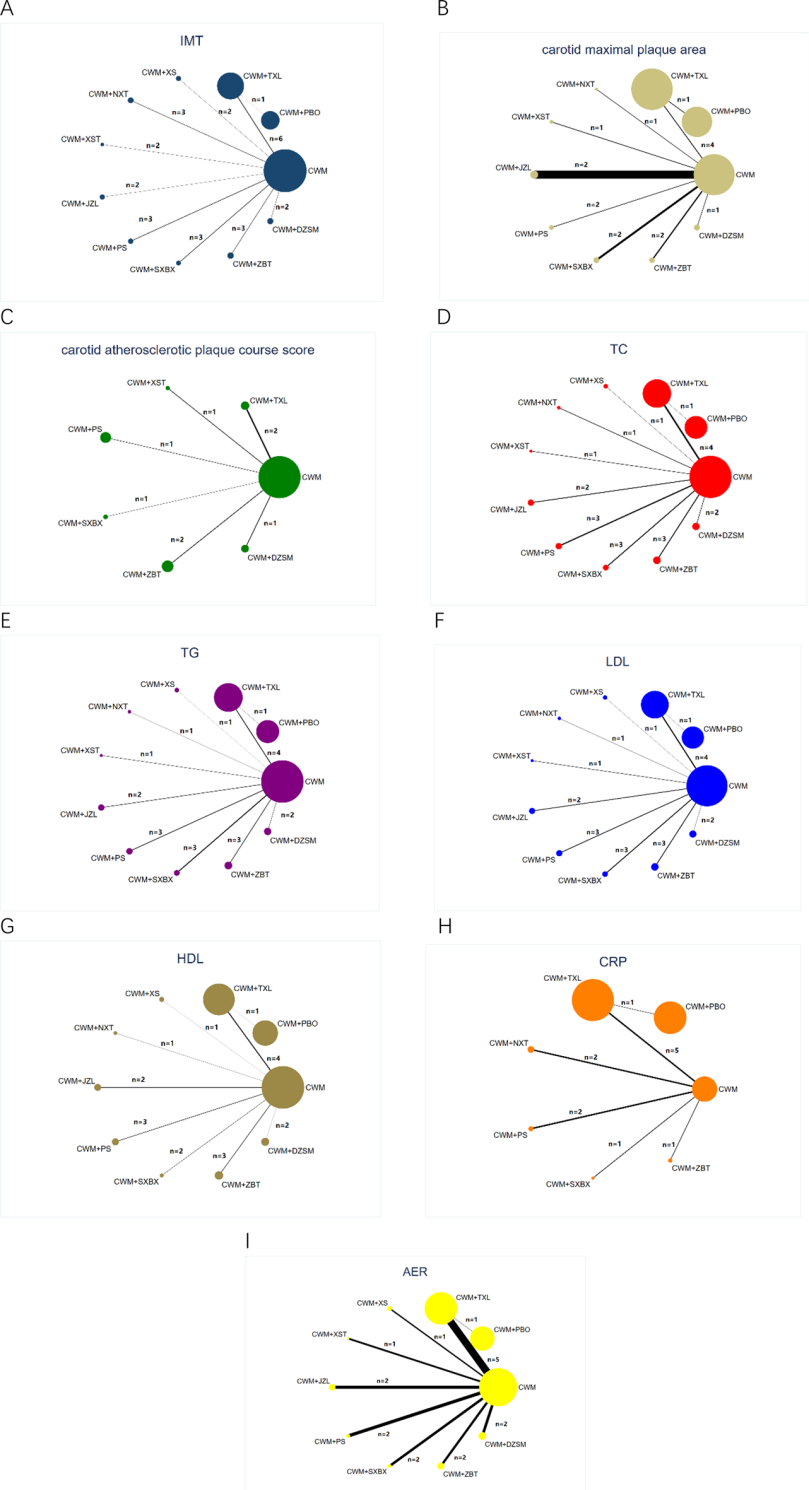
Network diagrams for different outcomes. **A**: IMT; **B**: carotid maximal plaque area; **C**: carotid atherosclerotic plaque course score; **D**: TC; **E**: TG; **F**: LDL; **G**: HDL; **H**: CRP; **I**: AER; *CWM* conventional western medicine, *PBO* placebo, *TXL* Tongxinluo capsule, *XS* Xiaoshuang granules/enteric capsule, *NXT* Naoxintong capsule, *XST* Xuesaitong capsule/soft capsule, *JZL* Jiangzhiling pill, *PS* Pushen capsule, *SXBX* Shexiang baoxin pill, *ZBT* Zhibitai, *DZSM* Dengzhan shengmai capsule, *IMT* carotid artery intimal- medial thickness, *TC* total cholesterol, *TG* Triglyceride, *LDL* low density lipoprotein, *HDL* high density lipoprotein, *CRP* C-reactive protein, *AER* adverse events rate. The width of the lines represents the proportion of the number of trials for each comparison with the total number of trials, and the size of the nodes represents the proportion of the number of randomized patients (sample sizes)

A total of 27 RCTs referred to the IMT of nine types of TCPMs and 11 types of interventions, including CWM + TXL vs. CWM + PBO (n = 1), CWM + TXL vs. CWM (n = 6), CWM + XS vs. CWM (n = 2), CWM + NXT vs. CWM (n = 3), CWM + XST vs. CWM (n = 2), CWM + JZL vs. CWM (n = 2), CWM + PS vs. CWM (n = 3), CWM + SXBX vs. CWM (n = 3), CWM + ZBT vs. CWM (n = 3), and CWM + DZSM vs. CWM (n = 2) (Table 2). Figure 4A presents the network evidence plot.

Compared to CWM, except for CWM + NXT [MD − 0.18 (95% CI − 0.39, 0.03)], CWM + XST [MD − 0.18 (95% CI − 0.43, 0.08)], CWM + PS [MD − 0.17 (95% CI: − 0.39, 0.04)] and CWM + DZSM [MD − 0.09 (95% CI − 0.34, 0.17)], other five TCPMs demonstrated a statistically significant effect in reducing the IMT. Accordingly, other interventions had no statistically significant difference. The details were shown in Table 4.

**Table 4:** Pairwise league table of IMT (lower− left quadrant) and carotid maximal plaque area (upper− right quadrant)

	Carotid maximal plaque area										
**IMT**	**CWM + TXL**	**–**	− 0.16 (− 12.71, 12.42)	0.95 (− 11.56, 13.52)	5.29 (− 5.13, 16.75)	− 1.37 (− 11.09, 8.39)	4.78 (− 4.72, 15.09)	0.50 (− 9.21, 10.22)	− 0.33 (− 12.91, 12.17)	− 2.31 (− 13.55, 9.05)	− 2.06 (− 7.67, 3.52)
	0.05 (− 0.25, 0.34)	**CWM + XS**	**–**	**–**	**–**	**–**	**–**	**–**	**−**	**–**	**–**
	− 0.04 (− 0.30, 0.22)	− 0.09 (− 0.41, 0.24)	**CWM + NXT**	1.12 (− 14.84, 16.91)	5.46 (− 8.71, 20.63)	− 1.21 (− 15.02, 12.58)	4.928 (− 8.56, 19.24)	0.68 (− 13.13, 14.38)	− 0.18 (− 16.041, 15.61)	− 2.16 (− 19.05, 14.76)	− 1.88 (− 13.15, 9.27)
	− 0.04 (− 0.33, 0.25)	− 0.09 (− 0.44, 0.27)	0.00 (− 0.33, 0.33)	**CWM + XST**	4.33 (− 9.76, 19.57)	− 2.31 (− 16.16, 11.51)	3.82 (− 9.57, 18.19)	− 0.44 (− 14.27, 13.28)	− 1.28 (− 17.18, 14.58)	− 3.25 (− 20.25, 13.63)	− 3.00 (− 14.23, 8.23)
	0.06 (− 0.24, 0.35)	0.01 (− 0.35, 0.37)	0.10 (− 0.23, 0.43)	0.10 (− 0.26, 0.45)	**CWM + JZL**	− 6.65 (− 19.49, 5.06)	− 0.50 (− 13.00, 11.75)	− 4.78 (− 17.63, 7.03)	− 5.61 (− 20.86, 8.43)	− 7.58 (− 23.82, 7.55)	− 7.36 (− 17.27, 1.61)
	− 0.04 (− 0.30, 0.22)	− 0.09 (− 0.42, 0.24)	− 0.01 (− 0.30, 0.30)	− 0.01 (− 0.34, 0.33)	− 0.10 (− 0.43, 0.23)	**CWM + PS**	6.15 (− 4.80, 17.95)	1.87 (− 9.39, 13.18)	1.04 (− 12.69, 14.79)	− 0.94 (− 15.89, 13.95)	− 0.68 (− 8.66, 7.26)
	0.05 (− 0.21, 0.31)	0.01 (− 0.33, 0.34)	0.09 (− 0.21, 0.40)	0.09 (− 0.24, 0.42)	− 0.01 (− 0.34, 0.32)	0.09 (− 0.21, 0.40)	**CWM + SXBX**	− 4.27 (− 16.10, 6.66)	− 5.12 (− 19.38, 8.27)	− 7.07 (− 22.47, 7.46)	− 6.83 (− 15.37, 0.91)
	− 0.01(− 0.26, 0.26)	− 0.05 (− 0.38, 0.28)	0.03 (− 0.26, 0.34)	0.03 (− 0.29, 0.37)	− 0.06 (− 0.39, 0.27)	0.04 (− 0.26, 0.34)	− 0.06 (− 0.35, 0.25)	**CWM + ZBT**	− 0.83 (− 14.55, 12.86)	− 2.82 (− 17.69, 12.05)	− 2.56 (− 10.51, 5.44)
	− 0.13 (− 0.43, 0.17)	− 0.18 (− 0.54, 0.18)	− 0.09 (− 0.42, 0.24)	− 0.09 (− 0.45, 0.27)	− 0.19 (− 0.55, 0.17)	− 0.09 (− 0.43, 0.25)	− 0.18 (− 0.52, 0.16)	− 0.13 (− 0.47, 0.20)	**CWM + DZSM**	− 1.99 (− 18.76, 14.96)	− 1.72 (− 12.99, 9.48)
	− 0.02 (− 0.37, 0.34)	− 0.06 (− 0.52, 0.39)	0.02 (− 0.41, 0.46)	0.02 (− 0.44, 0.48)	− 0.08 (− 0.54, 0.38)	0.03 (− 0.41, 0.47)	− 0.07 (− 0.50, 0.37)	− 0.01 (− 0.46, 0.42)	0.11 (− 0.35, 0.58)	**CWM + PBO**	0.27 (− 12.33, 12.80)
	− **0.22 (**− **0.36, **− **0.07)**	− **0.26 (**− **0.51, − 0.01)**	− 0.18 (− 0.39, 0.03)	− 0.18 (− 0.43, 0.08)	**− 0.27 (− 0.53, − 0.02)**	− 0.17 (− 0.39, 0.04)	**− 0.27 (− 0.48, − 0.05)**	**− 0.21 (− 0.43, − 0.01)**	− 0.09 (− 0.34, 0.17)	− 0.20 (− 0.58, 0.19)	**CWM**

*IMT* carotid artery medial-intimal thickness, *CWM* conventional western medicine, *PBO* placebo, *TXL* Tongxinluo capsule, *XS* Xiaoshuang granules/enteric capsule, *NXT* Naoxintong capsule, *XST* Xuesaitong capsule/soft capsule, *JZL* Jiangzhiling pill, *PS* Pushen capsule, *SXBX* Shexiang baoxin pill, *ZBT* Zhibitai, *DZSM* Dengzhan shengmai capsule.Data of comparisons for IMT and carotid maximal plaque area are SMD (95% CI). The 95% CI which don’t range across 0 favors the column–defining treatment and are showed in bold

**Fig. 5:**
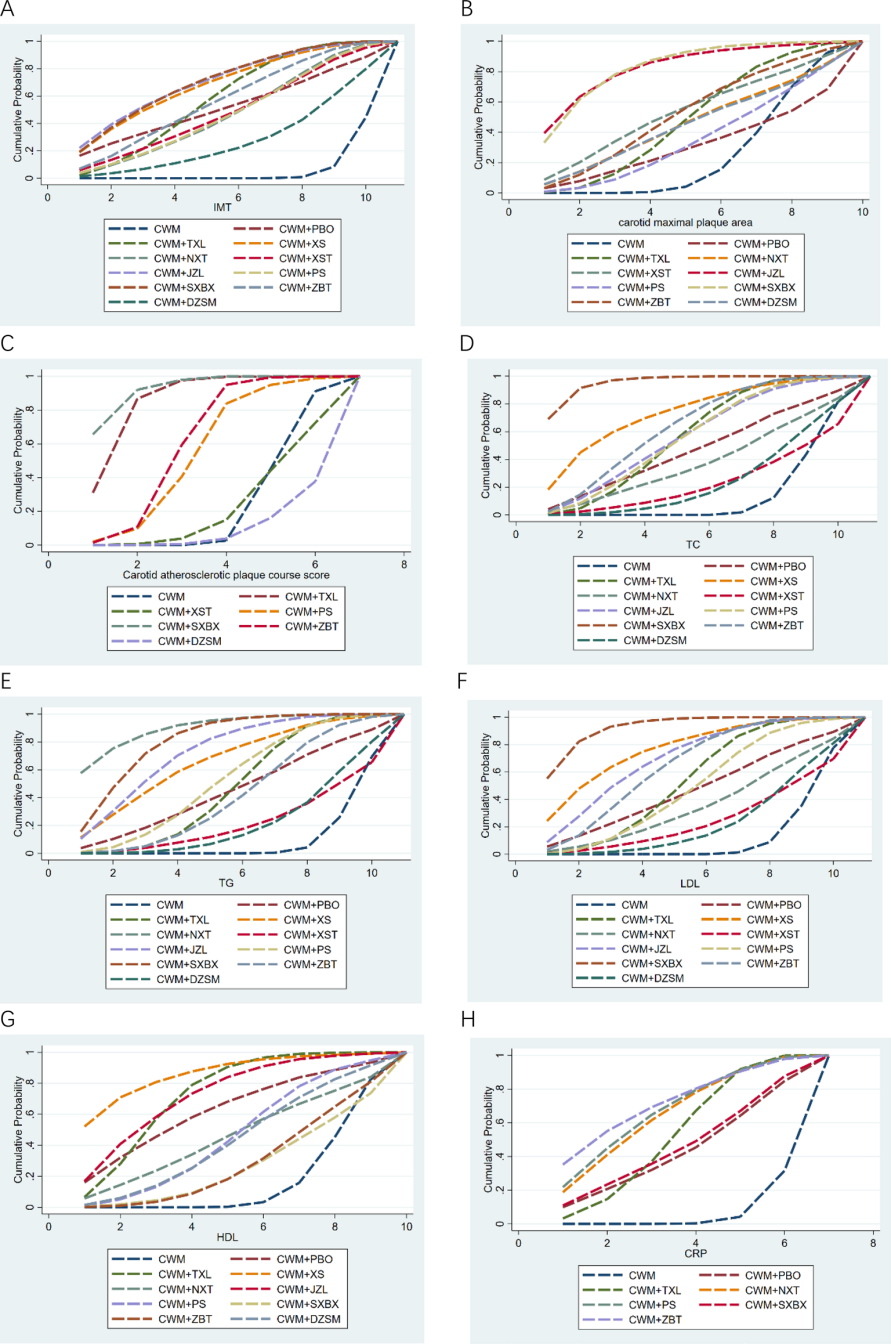
Surface under the cumulative ranking curve (SUCRA) plots for different outcomes. The vertical axis represents cumulative probabilities and the horizontal axis represents rank. **A**: IMT; **B**: carotid maximal plaque area; **C**: carotid atherosclerotic plaque course score; **D**: TC; **E**: TG; **F**: LDL; **G**: HDL; **H**: CRP; **I**: AER; *CWM* conventional western medicine, *PBO* placebo; *TXL* Tongxinluo capsule, *XS* Xiaoshuang granules/enteric capsule, *NXT* Naoxintong capsule, *XST* Xuesaitong capsule/soft capsule, *JZL* Jiangzhiling pill, *PS* Pushen capsule, *SXBX* Shexiang baoxin pill, *ZBT* Zhibitai, *DZSM* Dengzhan shengmai capsule, *IMT* carotid artery intimal-medial thickness, *TC* total cholesterol, *TG* Triglyceride, *LDL* low density lipoprotein, *HDL* high density lipoprotein, *CRP* C− reactive protein

According to the SUCRA probability results (Fig. 5A), CWM + JZL was likely the best intervention for reducing the IMT. Table 5 illustrates the detailed SUCRA and ranking probability. The interventions were ranked as follows: CWM + JZL (70.6%) > CWM + SXBX (70.5%) > CWM + XS (68.6%) > CWM + TXL (57.8%) > CWM + ZBT (56.5%) > CWM + PBO (51.7%) > CWM + XST (48.0%) > CWM + NXT (46.8%) > CWM + PS (46.8%) > CWM + DZSM (27.2%) >  > CWM (5.4%).

**Table 5:** Pairwise league table of TC (lower− left quadrant) and carotid atherosclerotic plaque course score (upper− right quadrant)

	Carotid atherosclerotic plaque course score										
**TC**	**CWM + TXL**	−	−	− 1.47 (− 4.12, 1.20)	−	− 1.69 (− 3.84, 0.47)	− 0.87 (− 3.51, 1.76)	− 1.00 (− 3.15, 1.16)	− 1.91 (− 4.56, 0.75)	−	− 1.45 (− 2.99, 0.09)
	0.31 (− 0.89, 1.52)	**CWM + XS**	**− **	**− **	**− **	**− **	**− **	**− **	**− **	**− **	**− **
	− 0.28 (− 1.53, 0.97)	− 0.59 (− 2.15, 0.96)	**CWM + NXT**	**− **	**− **	**− **	**− **	**− **	**− **	**− **	**− **
	− 0.55 (− 1.78, 0.68)	− 0.86 (− 2.40, 0.67)	− 0.27 (− 1.85, 1.29)	**CWM + XST**	−	− 0.22 (− 2.87, 2.42)	0.60 (− 2.47, 3.60)	0.47 (− 2.20, 3.09)	− 0.44 (− 3.50, 2.60)	−	0.02 (− 2.17, 2.18)
	− 0.01 (− 0.94, 0.94)	− 0.32 (− 1.64, 1.01)	0.28 (− 1.08, 1.64)	0.55 (− 0.79, 1.90)	**CWM + JZL**	**− **	**− **	**− **	**− **	**− **	**− **
	0.01 (− 0.84, 0.84)	− 0.31 (− 1.57, 0.93)	0.28 (− 1.02, 1.57)	0.56 (− 0.73, 1.82)	0.01 (− 1.01, 1.00)	**CWM + PS**	0.82 (− 1.81, 3.41)	0.69 (− 1.47, 2.81)	− 0.23 (− 2.86, 2.45)	−	0.24 (− 1.28, 1.74)
	0.75 (− 0.08, 1.58)	0.43 (− 0.80, 1.67)	1.02 (− 0.26, 2.31)	**1.30 (0.03, 2.57)**	0.75 (− 0.25, 1.73)	0.74 (− 0.14, 1.64)	**CWM + SXBX**	− 0.13 (− 2.75, 2.49)	− 1.03 (− 4.07, 2.03)	−	− 0.58 (− 2.71, 1.57)
	0.11 (− 0.72, 0.95)	− 0.21 (− 1.44, 1.04)	0.38 (− 0.89, 1.68)	0.66 (− 0.60, 1.93)	0.11 (− 0.88, 1.10)	0.10 (− 0.78, 1.01)	− 0.64 (− 1.52, 0.25)	**CWM + ZBT**	− 0.91 (− 3.56, 1.75)	−	− 0.45 (− 1.95, 1.08)
	− 0.45 (− 1.40, 0.48)	− 0.77 (− 2.09, 0.54)	− 0.17 (− 1.53, 1.17)	0.10 (− 1.25, 1.44)	− 0.45 (− 1.55, 0.61)	− 0.46 (− 1.45, 0.54)	− 1.20 (− 2.19, − 0.23)	− 0.56 (− 1.56, 0.41)	**CWM + DZSM**	**− **	0.46 (− 1.72, 2.62)
	− 0.13 (− 1.21, 0.94)	− 0.45 (− 2.06, 1.17)	0.15 (− 1.50, 1.79)	0.42 (− 1.22, 2.05)	− 0.13 (− 1.56, 1.30)	− 0.14 (− 1.49, 1.23)	− 0.88 (− 2.23, 0.48)	− 0.24 (− 1.61, 1.12)	0.32 (− 1.10, 1.75)	**CWM + PBO**	**− **
	**− 0.58 (− 1.14, − 0.03)**	− 0.90 (− 1.97, 0.17)	− 0.30 (− 1.43, 0.82)	− 0.03 (− 1.13, 1.07)	− 0.58 (− 1.35, 0.18)	− 0.59 (− 1.22, 0.06)	**− 1.33 (− 1.95, − 0.70)**	**− 0.69 (− 1.32, − 0.07)**	− 0.13 (− 0.88, 0.64)	− 0.45 (− 1.66, 0.75)	**CWM**

*TC* total cholesterol, *CWM* conventional western medicine, *PBO* placebo, *TXL* Tongxinluo capsule, *XS* Xiaoshuang granules/enteric capsule, *NXT* Naoxintong capsule, *XST* Xuesaitong capsule/soft capsule, *JZL* Jiangzhiling pill, *PS* Pushen capsule, *SXBX* Shexiang baoxin pill, *ZBT* Zhibitai, *DZSM* Dengzhan shengmai capsule.Data of comparisons for TC and carotid atherosclerotic plaque course score are SMD (95% CI). The 95% CI which don’t range across 0 favors the column-defining treatment and are showed in bold

#### Carotid maximal plaque area

A total of 16 RCTs referred to the carotid maximal plaque area of eight types of TCPMs and 10 types of interventions, including CWM + TXL vs. CWM + PBO (n = 1), CWM + TXL vs. CWM (n = 4), CWM + NXT vs. CWM (n = 1), CWM + XST vs. CWM (n = 1), CWM + JZL vs. CWM (n = 2), CWM + PS vs. CWM (n = 2), CWM + SXBX vs. CWM (n = 2), CWM + ZBT vs. CWM (n = 2), and CWM + DZSM vs. CWM (n = 1) (Table 2). Figure 4B presents the network evidence plot. All interventions had no statistically significant difference. The details were shown in Table 4.

According to the SUCRA probability results (Fig. 5B), CWM + SXBX was the most likely the best intervention for reducing the carotid maximal plaque area. Table 8 presents the detailed SUCRA and ranking probability. The ranking of interventions was as follows: CWM + SXBX (83.0%) > CWM + JZL (82.7%) > CWM + XST (53.1%) > CWM + ZBT (52.0%) > CWM + TXL (48.4%) > CWM + NXT (45.3%) > CWM + DZSM (44.7%) > CWM + PS (35.0%) > CWM + PBO (31.1%) > CWM (24.8%).

#### Carotid atherosclerotic plaque course score

Eight RCTs referred to the carotid atherosclerotic plaque Course score of six types of TCPMs and seven types of interventions, including CWM + TXL vs. CWM (n = 2), CWM + XST vs. CWM (n = 1), CWM + PS vs. CWM (n = 1), CWM + SXBX vs. CWM (n = 1), CWM + ZBT vs. CWM (n = 2), and CWM + DZSM vs. CWM (n = 1). (Table 2). Figure 4C presents the network evidence plot. All interventions had no statistically significant differences. The details were shown in Table 5**.**

According to the SUCRA probability results (Fig. 5C), CWM + XSBX was the most likely the best intervention for lowering the carotid atherosclerotic plaque Course score. Table 8 depicts the detailed SUCRA and ranking probability. The interventions were ranked as follows: CWM + SXBX (92.5%) > CWM + TXL (85.9%) > CWM + ZBT (61.0%) > CWM + PS (55.0%) > CWM (23.2%) > CWM + XST (22.7%) > CWM + DZSM (9.7%).

#### TC

A total of 21 RCTs referred to the TC of nine types of TCPMs and 11 types of interventions, including CWM + TXL vs. CWM + PBO (n = 1), CWM + TXL vs. CWM (n = 4), CWM + XS vs. CWM (n = 1), CWM + NXT vs. CWM (n = 1), CWM + XST vs. CWM (n = 1), CWM + JZL vs. CWM (n = 2), CWM + PS vs. CWM (n = 3), CWM + SXBX vs. CWM (n = 3), CWM + ZBT vs. CWM (n = 3), and CWM + DZSM vs. CWM (n = 2). (Table 2). Figure 4D presents the network evidence plot.

CWM + TXL [MD − 0.58 (95% CI − 1.14, − 0.03)], CWM + SXBX [MD − 1.33 (95% CI − 1.95, − 0.70)], and CWM + ZBT [MD − 0.69 (95% CI − 1.32, − 0.07)] had a statistically significant effect on lowering TC compared to CWM. CWM + SXBX [MD − 1.30 (95% CI − 2.57, − 0.03)] had a statistically significant effect on lowering TC compared to CWM + XST. Accordingly, other interventions had no statistically significant differences. The details were shown in Table 5.

According to the SUCRA probability results (Fig. 5D), CWM + XSBX was the most likely the best intervention for lowering TC. Table 8 indicates the detailed SUCRA and ranking probability. The 11 types of interventions were ranked as follows: CWM + SXBX (95.6%) > CWM + XS (73.6%) > CWM + ZBT (63.8%) > CWM + JZL (57.1%) > CWM + TXL (57.0%) > CWM + PS (56.3%) > CWM + PBO (46.9%) > CWM + NXT (37.9%) > CWM + DZSM (24.6%) > CWM + XST (23.2%) > CWM (14.0%).

#### TG

A total of 21 RCTs referred to the TG of nine types of TCPMs and 11 types of interventions, including CWM + TXL vs. CWM + PBO (n = 1), CWM + TXL vs. CWM (n = 4), CWM + XS vs. CWM (n = 1), CWM + NXT vs. CWM (n = 1), CWM + XST vs. CWM (n = 1), CWM + JZL vs. CWM (n = 2), CWM + PS vs. CWM (n = 3), CWM + SXBX vs. CWM (n = 3), CWM + ZBT vs. CWM (n = 3), and CWM + DZSM vs. CWM (n = 2) (Table 2). Figure 4E presents the network evidence plot.

CWM + NXT [MD − 0.76 (95% CI − 1.35, − 0.17)], CWM + JZL [MD − 0.52 (95% CI − 0.94, − 0.10)] and CWM + SXBX [MD − 0.59 (95% CI − 0.95, − 0.23)] had a statistically significant effect on lowering TG compared to CWM. Consequently, other interventions had no statistically significant differences. The details were shown in Table 6**.**

**Table 6:** Pairwise league table of LDL (lower− left quadrant) and TG (upper− right quadrant)

	**TG**										
**LDL**	**CWM + TXL**	0.17 (− 0.48, 0.83)	0.46 (− 0.20, 1.12)	− 0.25 (− 0.93, 0.43)	0.22 (− 0.30, 0.74)	0.04 (− 0.41, 0.50)	0.29 (− 0.17, 0.76)	− 0.05 (− 0.50, 0.41)	− 0.21 (− 0.72, 0.30)	− 0.02 (− 0.61, 0.57)	− 0.30 (− 0.60, 0.01)
	0.31 (− 0.60, 1.22)	**CWM + XS**	0.29 (− 0.54, 1.12)	− 0.42 (− 1.27, 0.43)	0.05 (− 0.67, 0.77)	− 0.13 (− 0.81, 0.55)	0.12 (− 0.57, 0.80)	− 0.22 (− 0.90, 0.46)	− 0.39 (− 1.10, 0.33)	− 0.19 (− 1.08, 0.69)	− 0.47 (− 1.05, 0.11)
	− 0.20 (− 1.11, 0.71)	− 0.51 (− 1.66, 0.64)	**CWM + NXT**	− 0.71 (− 1.56, 0.14)	− 0.24 (− 0.97, 0.49)	− 0.42 (− 1.10, 0.27)	− 0.17 (− 0.86, 0.52)	− 0.51 (− 1.19, 0.17)	− 0.67 (− 1.40, 0.05)	− 0.48 (− 1.37, 0.40)	**− 0.76 (− 1.35, − 0.17)**
	− 0.36 (− 1.30, 0.58)	− 0.67 (− 1.84, 0.49)	− 0.16 (− 1.33, 1.02)	**CWM + XST**	0.47 (− 0.28, 1.21)	0.29 (− 0.42, 1.00)	0.54 (− 0.17, 1.25)	0.20 (− 0.51, 0.90)	0.03 (− 0.71, 0.78)	0.23 (− 0.68, 1.13)	− 0.05 (− 0.66, 0.56)
	0.20 (− 0.52, 0.93)	− 0.11 (− 1.10, 0.90)	0.40 (− 0.60, 1.41)	0.56 (− 0.46, 1.60)	**CWM + JZL**	− 0.18 (− 0.72, 0.37)	0.07 (− 0.49, 0.62)	− 0.27 (− 0.82, 0.28)	− 0.43 (− 1.03, 0.16)	− 0.24 (− 1.03, 0.54)	**− 0.52 (− 0.94, − 0.10)**
	− 0.04 (− 0.66, 0.60)	− 0.34 (− 1.28, 0.60)	0.17 (− 0.78, 1.10)	0.33 (− 0.64, 1.29)	− 0.23 (− 0.99, 0.51)	**CWM + PS**	0.25 (− 0.25, 0.75)	− 0.09 (− 0.58, 0.40)	− 0.26 (− 0.80, 0.29)	− 0.06 (− 0.82, 0.68)	− 0.34 (− 0.69, 0.01)
	0.53 (− 0.11, 1.16)	0.22 (− 0.72, 1.16)	0.73 (− 0.22, 1.67)	0.90 (− 0.09, 1.85)	0.33 (− 0.44, 1.08)	0.57 (− 0.11, 1.24)	**CWM + SXBX**	− 0.34 (− 0.84, 0.16)	− 0.50 (− 1.05, 0.04)	− 0.31 (− 1.07, 0.44)	**− 0.59 (− 0.95, − 0.23)**
	0.12 (− 0.49, 0.76)	− 0.19 (− 1.11, 0.76)	0.32 (− 0.61, 1.28)	0.49 (− 0.47, 1.47)	− 0.07 (− 0.83, 0.69)	0.16 (− 0.50, 0.84)	− 0.41 (− 1.07, 0.29)	**CWM + ZBT**	− 0.16 (− 0.70, 0.37)	0.03 (− 0.72, 0.77)	− 0.25 (− 0.60, 0.09)
	− 0.33 (− 1.03, 0.38)	− 0.64 (− 1.62, 0.35)	− 0.13 (− 1.12, 0.87)	0.04 (− 0.98, 1.05)	− 0.53 (− 1.35, 0.29)	− 0.29 (− 1.03, 0.45)	**− 0.86 (− 1.60, − 0.11)**	− 0.45 (− 1.20, 0.27)	**CWM + DZSM**	0.19 (− 0.59, 0.97)	− 0.09 (− 0.50 0.33)
	− 0.07 (− 0.88, 0.73)	− 0.38 (− 1.59, 0.83)	0.13 (− 1.10, 1.35)	0.29 (− 0.95, 1.52)	− 0.27 (− 1.36, 0.80)	− 0.04 (− 1.07, 0.98)	− 0.61 (− 1.63, 0.42)	− 0.20 (− 1.23, 0.81)	0.26 (− 0.82, 1.31)	**CWM + PBO**	− 0.28 (− 0.94, 0.39)
	**− 0.43 (− 0.84, − 0.02)**	− 0.74 (− 1.54, 0.07)	− 0.23 (− 1.05, 0.58)	− 0.07 (− 0.91, 0.77)	**− 0.63 (− 1.22, − 0.05)**	− 0.40 (− 0.87, 0.08)	**− 0.96 (− 1.44, − 0.48)**	**− 0.56 (− 1.04, − 0.09)**	− 0.10 (− 0.67, 0.46)	− 0.36 (− 1.26, 0.55)	**CWM**

*LDL* low density lipoprotein, *TG* riglyceride, *CWM* conventional western medicine, *PBO* placebo, *TXL* Tongxinluo capsule, *XS* Xiaoshuang granules/enteric capsule, *NXT* Naoxintong capsule, *XST* Xuesaitong capsule/soft capsule, *JZL* Jiangzhiling pill, *PS* Pushen capsule, *SXBX* Shexiang baoxin pill, *ZBT* Zhibitai, *DZSM* Dengzhan shengmai capsule. Data of comparisons for LDL and TG are SMD (95% CI). The 95% CI which don’t range across 0 favors the column-defining treatment and are showed in bold

According to the SUCRA probability results (Fig. 5E), CWM + NXT was the most likely the best intervention for lowering the TG. Table 8 presents the detailed SUCRA and ranking probability. The interventions were ranked as follows: CWM + NXT (90.1%) > CWM + SXBX (81.1%) > CWM + JZL (72.7%) > CWM + XS (66.1%) > CWM + PS (52.5%) > CWM + TXL (47.0%) > CWM + PBO (44.7%) > CWM + ZBT (41.6%) > CWM + DZSM (22.2%) > CWM + XST (21.9%) > CWM (10.0%).

#### LDL

A total of 21 RCTs referred to the LDL of nine types of TCPMs and 11 types of interventions, including CWM + TXL vs. CWM + PBO (n = 1), CWM + TXL vs. CWM (n = 4), CWM + XS vs. CWM (n = 1), CWM + NXT vs. CWM (n = 1), CWM + XST vs. CWM (n = 1), CWM + JZL vs. CWM (n = 2), CWM + PS vs. CWM (n = 3), CWM + SXBX vs. CWM (n = 3), CWM + ZBT vs. CWM (n = 3), and CWM + DZSM vs. CWM (n = 2). (Table 2). Figure 4F presents the network evidence plot.

CWM + TXL [MD − 0.43 (95% CI − 0.84, − 0.02)], CWM + JZL [MD − 0.63 (95% CI − 1.22, − 0.05)], CWM + SXBX [MD − 0.96 (95% CI − 1.44, − 0.48)], and CWM + ZBT [MD − 0.56 (95% CI − 1.04, − 0.09)] has a statistically significant effect on lowering LDL compared to CWM. CWM + SXBX [MD − 0.86 (95% CI − 1.60, − 0.11)] had a statistically significant effect on lowering LDL compared to CWM + DZSM. Therefore, other interventions had no statistically significant difference. The details were shown in Table 6**.**

According to the SUCRA probability results (Fig. 5F), CWM + SXBX was the most likely the best intervention for lowering the LDL. Table 8 depicts the detailed SUCRA and ranking probability. The interventions were ranked as follows: CWM + SXBX (92.6%) > CWM + XS (76.9%) > CWM + JZL (69.9%) > CWM + ZBT (64.4%) > CWM + TXL (53.5%) > CWM + PS (49.1%) > CWM + PBO (47.0%) > CWM + NXT (35.9%) > CWM + XST (24.9%) > CWM + DZSM (23.5%) > CWM (12.3%).

#### HDL

A total of 19 RCTs referred to the HDL of eight types of TCPMs and 10 types of interventions, including CWM + TXL vs. CWM + PBO (n = 1), CWM + TXL vs. CWM (n = 4), CWM + XS vs. CWM (n = 1), CWM + NXT vs. CWM (n = 1), CWM + JZL vs. CWM (n = 2), CWM + PS vs. CWM (n = 3), CWM + SXBX vs. CWM (n = 2), CWM + ZBT vs. CWM (n = 3), and CWM + DZSM vs. CWM (n = 2). (Table 2). Figure 4G presents the network evidence plot.

CWM + TXL [MD 0.34 (95% CI: 0.05, 0.64)] had a statistically significant effect on raising HDL compared to CWM. Thus, no statistically significant difference existed between the other interventions. The details were shown in Table 7.

**Table 7:** Pairwise league table of HDL (lower-left quadrant) and CRP (upper-right quadrant)

	CRP										
HDL	**CWM + TXL**	–	0.19 (− 1.14, 1.48)	–	–	0.23 (− 1.11, 1.51)	− 0.10 (− 1.81, 1.54)	0.31 (− 1.39, 1.94)	–	− 0.14 (− 1.60, 1.32)	− 0.67 (− 1.45, 0.04)
	− 0.18 (− 0.81, 0.46)	**CWM + XS**	−	–	–	–	–	–	–	–	–
	0.18 (− 0.47, 0.83)	0.36 (− 0.44, 1.17)	**CWM + NXT**	–	–	0.04 (− 1.49, 1.57)	− 0.30 (− 2.15, 1.55)	0.11 (− 1.73, 1.96)	–	− 0.33 (− 2.26, 1.66)	− 0.87 (− 1.95, 0.21)
	–	–	–	**CWM + XST**	–	–	–	–	–	−	−
	− 0.02 (− 0.55, 0.48)	0.16 (− 0.56, 0.84)	− 0.20 (− 0.93, 0.49)	–	**CWM + JZL**	–	–	–	–	−	−
	0.18 (− 0.27, 0.62)	0.36 (− 0.30, 1.01)	− 0.01 (− 0.67, 0.66)	–	0.20 (− 0.32, 0.75)	**CWM + PS**	− 0.34 (− 2.20, 1.51)	0.07 (− 1.78, 1.91)	–	− 0.37 (− 2.31, 1.61)	− 0.91 (− 1.99, 0.18)
	0.30 (− 0.20, 0.80)	0.48 (− 0.22, 1.17)	0.12 (− 0.58, 0.82)	–	0.32 (− 0.25, 0.92)	0.12 (− 0.40, 0.64)	**CWM + SXBX**	0.41 (− 1.70, 2.53)	–	− 0.04 (− 2.21, 2.23)	− 0.57 (− 2.07, 0.94)
	0.29 (− 0.16, 0.73)	0.46 (− 0.20, 1.12)	0.10 (− 0.56, 0.76)	–	0.31 (− 0.21, 0.86)	0.10 (− 0.36, 0.58)	− 0.02 (− 0.54, 0.51)	**CWM + ZBT**	–	− 0.44 (− 2.62, 1.79)	− 0.98 (− 2.47, 0.51)
	0.19 (− 0.31, 0.69)	0.37 (− 0.32, 1.06)	0.01 (− 0.69, 0.71)	–	0.21 (− 0.35, 0.81)	0.01 (− 0.51, 0.53)	− 0.11 (− 0.68, 0.46)	− 0.09 (− 0.61, 0.43)	**CWM + DZSM**	–	–
	0.04 (− 0.52, 0.61)	0.22 (− 0.63, 1.07)	− 0.14 (− 0.99, 0.72)	–	0.06 (− 0.68, 0.84)	− 0.14 (− 0.85, 0.58)	− 0.26 (− 1.01, 0.50)	− 0.24 (− 0.96, 0.48)	− 0.15 (− 0.90, 0.60)	**CWM + PBO**	− 0.53 (− 2.21, 1.07)
	**0.34 (0.05, 0.64)**	0.52 (− 0.05, 1.08)	0.16 (− 0.41, 0.73)	–	0.36 (− 0.04, 0.80)	0.16 (− 0.17, 0.50)	0.04 (− 0.36, 0.44)	0.06 (− 0.28, 0.39)	0.15 (− 0.25, 0.55)	0.30 (− 0.34, 0.93)	**CWM**

*HDL* high density lipoprotein, *CRP* C-reactive protein, *CWM* conventional western medicine, *PBO* placebo, *TXL* Tongxinluo capsule, *XS* Xiaoshuang granules/enteric capsule, *NXT* Naoxintong capsule, *XST* Xuesaitong capsule/soft capsule, *JZL* Jiangzhiling pill, *PS* Pushen capsule, *SXBX* Shexiang baoxin pill, *ZBT* Zhibitai, *DZSM* Dengzhan shengmai capsule. Data of comparisons for HDL and CRP are SMD (95% CI). The 95% CI which don’t range across 0 favors the column− defining treatment and are showed in bold

According to the SUCRA probability results (Fig. 5G), CWM + XS was the most likely the best intervention for improving HDL. Table 8 illustrates the detailed SUCRA and ranking probability. The interventions were ranked as follows: CWM + XS (86.1%) > CWM + JZL (72.9%) > CWM + TXL (72.9%) > CWM + PBO (62.4%) > CWM + PS (45.6%) > CWM + NXT (45.2%) > CWM + DZSM (43.1%) > CWM + ZBT (28.6%) > CWM + SXBX (26.8%) > CWM (16.4%).

**Table 8:** Surface under the cumulative ranking curve and ranking probability of different Chinese patent medicines on each outcome

Treatment	IMT		Carotid maximal plaque area		Carotid atherosclerotic plaque course score		TC		TG		LDL		HDL		CRP	
	SUCRA	Rank	SUCRA	Rank	SUCRA	Rank	SUCRA	Rank	SUCRA	Rank	SUCRA	Rank	SUCRA	Rank	SUCRA	Rank
CWM	5.40%	11	24.80%	10	23.20%	5	14.00%	11	10.00%	11	12.30%	11	16.40%	10	6.00%	7
CWM + PBO	51.70%	6	31.10%	9	–	–	46.90%	7	44.70%	7	47.00%	7	62.40%	4	42.80%	6
CWM + TXL	57.80%	4	48.40%	5	**85.9%**	**2**	57.00%	5	47.00%	6	53.50%	5	72.90%	3	52.30%	4
CWM + XS	68.60%	3	–	–	–	–	73.60%	2	66.10%	4	76.90%	2	**86.10%**	**1**	–	–
CWM + NXT	46.80%	8	45.30%	6	–	–	37.90%	8	**90.10%**	**1**	35.90%	8	45.20%	6	64.90%	3
CWM + XST	48.00%	7	53.10%	3	22.70%	6	23.20%	10	21.90%	10	24.90%	9	−	−	−	−
CWM + JZL	**70.60%**	**1**	82.70%	2	–	–	57.10%	4	72.70%	3	69.90%	3	72.90%	2	**− **	−
CWM + PS	46.80%	9	35.00%	8	55.00%	4	56.30%	6	52.50%	5	49.10%	6	45.60%	5	67.00%	2
CWM + SXBX	70.50%	2	**83.00%**	**1**	92.50%	1	**95.60%**	**1**	81.10%	2	**92.60%**	**1**	26.80%	9	45.70%	5
CWM + ZBT	56.50%	5	52.00%	4	61.00%	3	63.80%	3	41.60%	8	64.40%	4	28.60%	8	**71.30%**	**1**
CWM + DZSM	27.20%	10	44.70%	7	9.70%	7	24.60%	9	22.20%	9	23.50%	10	43.10%	7	–	–

*CWM* conventional western medicine, *PBO* placebo, *TXL* Tongxinluo capsule, *XS* Xiaoshuang granules/enteric capsule, *NXT* Naoxintong capsule, *XST* Xuesaitong capsule/soft capsule, *JZL* Jiangzhiling pill, *PS* Pushen capsule, *SXBX* Shexiang baoxin pill, *ZBT* Zhibitai, *DZSM* Dengzhan shengmai capsule, *IMT* carotid artery intimal-medial thickness, *TC* total cholesterol, *TG* Triglyceride, *LDL* low density lipoprotein, *HDL* high density lipoprotein, *CRP* C-reactive protein

#### CRP

A total of 11 RCTs referred to the CRP of five types of TCPMs and seven types of interventions, including CWM + TXL vs. CWM + PBO (n = 1), CWM + TXL vs. CWM (n = 5), CWM + NXT vs. CWM (n = 2), CWM + PS vs. CWM (n = 2), CWM + SXBX vs. CWM (n = 1), and CWM + ZBT vs. CWM (n = 1). (Table 2). Figure 4H presents the network evidence plot. All interventions had no statistically significant difference. The details were shown in Table 7**.**

According to the SUCRA probability results (Fig. 5H), CWM + ZBT was the most likely the best intervention for lowering the CRP. Table 8 presents the detailed SUCRA and ranking probability. The interventions were ranked as follows: CWM + ZBT (71.3%) > CWM + PS (67.0%) > CWM + NXT (64.9%) > CWM + TXL (52.3%) > CWM + SXBX (45.7%) > CWM + PBO (42.8%) > CWM (6.0%).

#### Safety

A total of 18 RCTs reported the number of the AER of eight types of TCPMs and 10 types of interventions, including CWM + TXL vs. CWM + PBO (n = 1), CWM + TXL vs. CWM (n = 5), CWM + XS vs. CWM (n = 1), CWM + XST vs. CWM (n = 1), CWM + JZL vs. CWM (n = 2), CWM + PS vs. CWM (n = 2), CWM + SXBX vs. CWM (n = 2), CWM + ZBT vs. CWM (n = 2), and CWM + DZSM vs. CWM (n = 2) (Table 2). Figure 4I presents the network evidence plot.

Four studies reported no adverse reactions in the experimental and control groups, while the remaining 14 studies reported 204 cases of adverse reactions. Adverse events included gastrointestinal reactions, such as nausea, discomfort, indigestion, abdominal distension, pain, and diarrhea. Autonomic nervous dysfunction symptoms had dizziness, headache, rash, myalgia, mild hepatic or renal insufficiency, bleeding, and delayed PT. However, most resolved spontaneously without special treatment. The detailed list of adverse reactions was shown in Table 9.

**Table 9:** Occurrence of adverse reactions

Treatment	Study ID	AEs	Adverse reactions		Response
			Treatment group	Control group	
CWM + TXL vs. CWM + PBO	**Zhang M2019**	100	Hepatic insufficiency (seven cases), renal insufficiency (one case), headache (10 cases), stomach discomfort (24 cases), abdominal pain and diarrhea (four cases), bleeding or delayed PT (eight cases), allergic rash or asthma (one case)	Hepatic insufficiency (five cases), renal insufficiency (two cases), headache (11 cases), stomach discomfort (14 cases), abdominal pain and diarrhea (six cases), bleeding or delayed PT (two cases), allergic rash or asthma (one case), mental disorders (one case), insomnia (three cases)	–
CWM + TXL vs. CWM	**Ni ZX2017**	7	Gastrointestinal reactions (two cases)	Gastrointestinal reactions (three cases) and mild liver function abnormalities (two cases)	–
	**Wang SY2016**	0	0	0	–
	**Zhu D2016**	1	Mild nausea (one case)	0	–
	**Huang ZJ2014**	13	Nausea and abdominal pain (five cases), dizziness and headache (two cases), skin itch (one case)	Gastrointestinal discomfort (two cases), dizziness, and headache (three cases)	–
	**Wang H2011**	1	0	Mild liver function abnormalities (one case)	After liver protection and other symptomatic treatment, liver function returned to normal
CWM + XS vs. CWM	**Liu HJ2018**	3	Mild liver function abnormalities (one case)	Mild liver function abnormalities (two cases)	–
CWM + XST vs. CWM	**Jiang XP2021**	1	0	Mild liver function abnormalities (one case)	–
CWM + JZL vs. CWM	**Zhang XL2017**	6	Mild liver function abnormalities (four cases)	Mild liver function abnormalities (two cases)	–
	**Zhang J2016**	0	0	0	–
CWM + PS vs. CWM	**Wang HF2019**	0	0	0	–
	**Liu J2017**	11	Gastrointestinal discomfort (two cases), myalgia (one case), and mild liver function abnormalities (two cases)	Skin itch (one case), gastrointestinal discomfort (one case), myalgia (two cases), and mild liver function abnormalities (two cases)	Two groups of patients with myalgia and mild liver function abnormalities requested a change of medication and abandoned treatment
CWM + SXBX vs. CWM	**WangBJ2018**	2	Mild liver function abnormalities (two cases)		After liver protection and other symptomatic treatment, liver function returned to normal
	**JiaLW2004**	2	0	Mild upper abdominal discomfort (two cases)	–
CWM + ZBT vs. CWM	**XiaYM2021**	23	Abdominal pain and distention (three cases), myalgia (one case), mild liver function abnormalities (one case)	Abdominal pain and distention (five cases), headache (three cases), myalgia (three cases), mild liver function abnormalities (four cases), myocardial enzyme injury (two cases), rash (one case)	–
	**PengL2020**	2	Gastrointestinal discomfort (two cases)		It resolved spontaneously without special treatment
CWM + DZSM vs. CWM	**XuS2022**	32	Gastrointestinal discomfort (four cases), tumor (one case), skin rash (one case), myalgia (four cases), herpes zoster (one case)	Bleeding event (four cases), gastrointestinal discomfort (12 cases), tumor (one case), myalgia (three cases), acute cholecystitis (one case)	–
	**HuangP2018**	0	0	0	

*CWM* conventional western medicine, *PBO* placebo, *TXL* Tongxinluo capsule, *XS* Xiaoshuang granules/enteric capsule, *NXT* Naoxintong capsule, *XST* Xuesaitong capsule/soft capsule, *JZL* Jiangzhiling pill, *PS* Pushen capsule, *SXBX* Shexiang baoxin pill, *ZBT* Zhibitai, *DZSM* Dengzhan shengmai capsule

#### Inconsistency test

No closed loops were found in the NMA due to the lack of direct comparison of TCPMs. The inconsistency test could not be carried out. Hence, the results were analyzed using a consistency model.

#### Publication bias

**Fig. 6:**
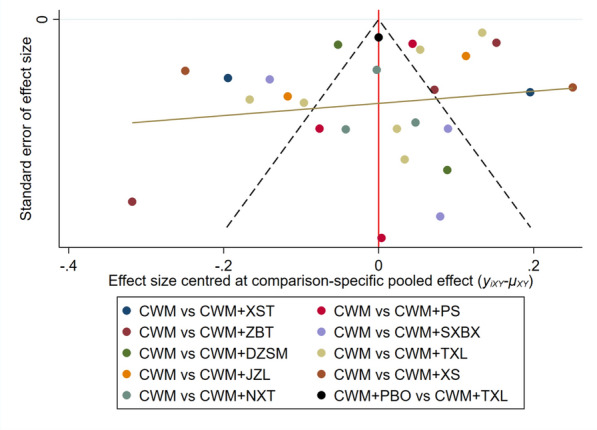
Funnel plot of IMT. *CWM* conventional western medicine, *PBO* placebo, *TXL* Tongxinluo capsule, *XS* Xiaoshuang granules/enteric capsule, *NXT* Naoxintong capsule, *XST* Xuesaitong capsule/soft capsule, *JZL* Jiangzhiling pill, *PS* Pushen capsule, *SXBX* Shexiang baoxin pill, *ZBT* Zhibitai, Dengzhan shengmai capsule

IMT is the leading indicator for publishing the results of the evaluation applications. The comparison− adjusted funnel plots were plotted to test the publication bias of IMT. When the points in the funnel chart are symmetrical based on the position of the centerline, presenting that there is no publication bias. Figure 6 depicts that the points in the funnel chart are asymmetrical along the center line, indicating the potential presence of publication bias favoring CWM + TCPMs in reducing IMT, as compared to CWM and CWM + PBO.

## Discussion

OMT, a pharmacotherapy regimen based on statins, is an important non− invasive treatment for CAP. The clinical efficacy of OMT can be improved by adding complementary and alternative medicines [54]. In our study, this NMA was based on 27 RCT trials with 4131 patients with CAP. We compared the efficacy and safety of nine kinds of TCPMs, including JZL, SXBX, TXL, ZBT, XS, XST, NXT, PS, and DZSM, combined with CWM with or without placebo of TCPM for improving IMT, carotid maximal plaque area, carotid atherosclerotic plaque Course score, serum lipid levels, and CRP. Pairwise meta− analyses demonstrated that CWM + TCPM was superior to CWM in the treatment of CAP. This study revealed that CWM + JZL was the most likely the best intervention for reducing IMT, and CWM + SXBX exhibited the highest effective intervention for reducing carotid maximal plaque area, and atherosclerotic plaque Course score. Lipids and inflammatory factors contribute to an increase in CAP volume and vulnerability [55]. The guideline has recommended that LDL− C and CRP are independent risk factors for atherosclerosis and play important roles in the primary and secondary prevention of atherosclerosis [56]. Our study suggested that CWM + XSBX was superior to other TCPMs in decreasing the TC and LDL levels. CWM + NXT and CWM + XS were superior to other TCPMs in reducing TG and increasing HDL, respectively. ​CWM + ZBT was the most likely the best intervention for lowering the CRP. Together, these results implied that CWM + TCPM may be a more effective intervention for patients with CAP than using CWM alone. Of the TCPMs included, SXBX was among the most effective in reducing carotid maxima, atherosclerotic plaque score, TC and LDL levels, and had a more comprehensive advantage. However, the efficacy of XSBX also needs to be evaluated through high− quality, large, double− blind, randomized controlled trials. XSBX still needs to be used with caution. No serious adverse events were reported in the CWM + TCPM and CWM groups. However, adverse events were poorly reported (18/27) in the included studies, and the safety of TCPMs needs further investigation.

Numerous pharmacological studies have also found that TCPMs could improve CAP through multiple targets and signaling pathways. JZL, which traditionally removes dampness and dissolves phlegm, was the best intervention for reducing IMT in this study. *Crataegus pinnatifida* Bunge, the essential herb of JZL, has anti− atherosclerotic effects by lowering blood lipids, inhibiting oxidative and inflammation, and protecting vascular endothelium [57]. According to TCM theory, XSBX has the traditional functions of resuscitation with aromatics, modifying Qi, and activating circulation. XSBX was the optimal drug for reducing the carotid maximal plaque area compared to the other eight CPMs. A pharmacological study also demonstrated that XSBX could markedly decrease atherosclerotic plaque size by inhibiting the arterial wall's inflammation response and lipid accumulation [58]. XSBX reduced the inflammation pathways by increasing Mfn2 and decreasing the phosphorylation of p38, JNK, and NF− κB levels. XSBX inhibited lipid influx by reducing SR− A and LOX− 1 and increased lipid efflux by promoting LXRα, ABCA1, and ABCG1. Additionally, XSBX could activate macrophages to improve endothelial cell proliferation, migration, and tubule formation and regulate PI3K/Akt and MAPK/Erk1/2 signaling pathways, thereby promoting angiogenesis [59]. Plaque thickness is the principal predictor of carotid stenosis risk. TXL, which traditionally promotes circulation to remove meridional obstructions, was optimal for treating carotid atherosclerotic plaque Course score in nine TCPMs. A study discovered that TXL could inhibit arterial intimal proliferation by reducing the LOX− 1 and improving blood lipids [60]. Moreover, several studies have exposed that TXL could improve plaque stability by inhibiting ROS expression and increasing the relative abundance of *Alistipes* in the gut microbiome [61].

This NMA study had several strengths. First, this study was the first to evaluate the comparative efficacy and safety of TCPMs for CAP and to guide optimal medication in a clinical setting. Second, this study set strict inclusion criteria and excluded RCTs with incorrect randomization methods, ensuring methodological quality. Finally, the ranking of TCPMs contributed to the formulation of clinical medication plans.

However, this study still has some limitations. First, the overall quality of the studies included was limited because most studies did not report the allocation concealment and blinding in detail. Additionally, clinical heterogeneity may have occurred due to the diversity of CWM and the various TCPMs dosage and duration, and these results should be interpreted with caution. Finally, assuming that the studies included were mainly conducted among Chinese populations, the external adaptability of the results would be restricted when applied for reference in populations of different countries and regions.

## Conclusions

This study aims to evaluate the efficacy of TCPMs in treating CAP based on the characteristics of carotid plaque, blood lipids, inflammatory markers, and adverse reactions to guide the clinical medication of CAP more accurately. CWM + JZL was the most effective in reducing IMT. CWM + SXBX was the most effective in reducing carotid maximal plaque area, and atherosclerotic plaque Course score. CWM + XSBX also significantly reduced TC and LDL levels and outperformed other CPMs. CWM + XSBX may be considered an effective intervention for the treatment of CAP. However, further direct comparisons are warranted. This study provides a more accurate selection of TCPMs in CAP therapy, which may help improve drug regimens of OMT by supplementing complementary and alternative drugs. More adequately powered, well− designed clinical trials to increase the quality of the available evidence are still needed in the future due to several limitations.

### Supplementary Information

**Additional file 1.** Searching strategies.

## Data Availability

All data supporting this systematic review and meta− analysis are from previously reported studies and datasets, which have been cited.

## Abbreviations

AER: Adverse events rate
CAP: Carotid atherosclerotic plaque
CAS: Carotid stent placement
CEA: Carotid endarterectomy
CRP: C− reactive protein
CWM: Conventional western medicine
DZSM: Dengzhan shengmai capsule
HDL: High density lipoprotein
IMT: Carotid artery intimal-medial thickness
JZL: Jiangzhiling pill
LDL: Low density lipoprotein
NMA: Network meta-analysis
NXT: Naoxintong capsule
OMT: Optimal drug therapy
PBO: Placebo
PS: Pushen capsule
RCTs: Randomized controlled trials
SUCRA: Surface under the cumulative ranking
SXBX: Shexiang baoxin pill
TC: Total cholesterol
TCM: Traditional Chinese medicine
TCPMs: Traditional Chinese patent medicine
TG: Triglyceride
TXL: Tongxinluo capsule
XS: Xiaoshuang granules/enteric capsule
XST: Xuesaitong capsule/soft capsule
ZBT: Zhibitai
