# Supplementary material for: Comparative efficacy of Chinese patent medicines in patients with carotid atherosclerotic plaque: a Bayesian network meta− analysis
Source: Chin Med. 2023 Nov 20;18:152. doi: 10.1186/s13020-023-00850-5 (PMC10662928; doi:10.1186/s13020-023-00850-5)
Supplement: Supplementary file 1 — Additional file 1. Searching strategies. [file 13020_2023_850_MOESM1_ESM.doc]

**Additional file**

**Title:** Comparative efficacy of Chinese patent medicines in patients with carotid atherosclerotic plaque: A Bayesian network meta-analysis

**AUTHOR：**Wenquan Su^a, 1^, Xiaolong Xie^a, 1^, Jiping Zhao^a^, Qinhua Fan^a^, Naijia Dong^a^, Qingxiao Li^a^, Yawei Du^a^, *, and Shengxian Wu^a^,*

^a^ Dongzhimen Hospital, Beijing University of Chinese Medicine, Beijing 100700, China

*Correspondence: shengxianwu@126.com (S.X. Wu); yaweidu@hotmail.com (Y.W. Du)

^1^ Wenquan Su and Xiaolong Xie contributed equally to this work.

**Contents**

### Supplementary appendix I

Searching strategies

**Supplementary appendix I: Searching strategies**

**Searching Strategies (CNKI)**

**TKA =(‘中医’ + ‘中药’ + ‘方药’ + ‘草药’+ ‘中西医’+ ‘中成药’+ ‘传统医学’+ ‘结合医学’+ ‘替代医学’+ ‘补充替代医学’) AND TKA=( ‘颈动脉粥样硬化’+‘颈动脉斑块’) AND TKA=（‘随机对照’+‘随机’+‘对照’+‘RCT’+‘安慰剂’ ）**

**Searching Strategies (WanFang)**

**(题名或关键词:(“中医” or “中药” or “方药” or “草药” or “中西医”or “中成药”or “传统医学”or “结合医学”or “替代医学”or “补充替代医学”) and 题名或关键词: (“颈动脉粥样硬化” or “颈动脉斑块”) and 题名或关键词: (“随机对照” or “随机” or “对照” or “安慰剂” or “RCT”)) or(摘要:( “中医” or “中药” or “方药” or “草药” or “中西医”or “中成药”or “传统医学”or “结合医学”or “替代医学”or “补充替代医学”) and 摘要:( “颈动脉粥样硬化” or “颈动脉斑块”) and 摘要:( “随机对照” or “随机” or “对照” or “安慰剂” or “RCT”))**

**Searching Strategies (VIP Database)**

**（（M=（中医 or 中药 or 方药 or 草药 or 中西医or 中成药 or 传统医学 or 结合医学 or 替代医学 or 补充替代医学）） AND （M=（颈动脉粥样硬化 or 颈动脉斑块）） AND （M= （随机对照 or 随机 or 对照 or 安慰剂 or RCT））） OR （（R=（中医 or 中药 or 方药 or 草药 or 中西医or 中成药 or 传统医学 or 结合医学 or 替代医学 or 补充替代医学）） AND （R=（颈动脉粥样硬化 or 颈动脉斑块）） AND （R= （随机对照 or 随机 or 对照 or 安慰剂 or RCT）））**

**Searching Strategies (CBM)**

**((颈动脉粥样硬化[标题:智能] OR 颈动脉粥样硬化[标题:智能]) AND (中医[标题:智能] OR 中药 [标题:智能] OR 方药 [标题:智能] OR 草药 [标题:智能] OR 中西医 [标题:智能] OR 中成药 [标题:智能] OR 传统医学 [标题:智能] OR 结合医学 [标题:智能] OR 替代医学 [标题:智能] OR 补充替代医学 [标题:智能]) AND (随机对照 [标题:智能] OR 随机 [标题:智能] OR 对照 [标题:智能] OR 安慰剂 [标题:智能] OR RCT [标题:智能])) OR ((颈动脉粥样硬化 [摘要:智能] OR 颈动脉粥样硬化 [摘要:智能]) AND (中医 [摘要:智能] OR 中药 [摘要:智能] OR 方药 [摘要:智能] OR 草药 [摘要:智能] OR 中西医 [摘要:智能] OR 中成药 [摘要:智能] OR 传统医学 [摘要:智能] OR 结合医学 [摘要:智能] OR 替代医学[摘要:智能] OR 补充替代医学 [摘要:智能]) AND (随机对照 [摘要:智能] OR 随机 [摘要:智能] OR 对照 [摘要:智能] OR 安慰剂 [摘要:智能] OR RCT [摘要:智能]))**

**Searching Strategies (PubMed)**

| **Search number** | **Query** |
| --- | --- |
| **1** | ("Carotid Stenosis"[Mesh]) OR (Carotid Stenoses) OR (Stenoses, Carotid) OR (Stenosis, Carotid) OR (Carotid Artery Narrowing) OR (Artery Narrowing, Carotid) OR (Artery Narrowings, Carotid) OR (Carotid Artery Narrowings) OR (Narrowings, Carotid Artery) OR (Carotid Artery Stenosis) OR (Artery Stenoses, Carotid) OR (Artery Stenosis, Carotid) OR (Carotid Artery Stenoses) OR (Stenosis, Carotid Artery) OR (Internal Carotid Artery Stenosis) OR (External Carotid Artery Stenosis) OR (Stenosis, External Carotid Artery) OR (Carotid Artery Plaque) OR (Artery Plaque, Carotid) OR (Artery Plaques, Carotid) OR (Carotid Artery Plaques) OR (Plaque, Carotid Artery) OR (Plaques, Carotid Artery) OR (Carotid Ulcer) OR (Ulcer, Carotid) |
| **2** | ("Medicine, Chinese Traditional"[Mesh]) OR (Zhong Yi Xue) OR (Chung I Hsueh) OR (Hsueh, Chung I) OR (Chinese Medicine, Traditional) OR (Chinese Traditional Medicine) OR (Traditional Chinese Medicine) OR (Traditional Tongue Diagnosis) OR (Tongue Diagnoses, Traditional) |
| **3** | (randomized controlled trial[Title/Abstract]) OR (randomized[Title/Abstract]) OR (placebo[Title/Abstract]) |
| **4** | #1 AND #2 AND #3 |

**Searching Strategies (Cochrane)**

| **Search number** | **Query** |
| --- | --- |
| **1** | (Carotid Stenosis):ab,ti,kw OR (Carotid Stenoses):ab,ti,kw OR (Stenoses, Carotid):ab,ti,kw OR (Stenosis, Carotid):ab,ti,kw OR (Carotid Artery Narrowing):ab,ti,kw OR (Artery Narrowing, Carotid):ab,ti,kw OR (Artery Narrowings, Carotid):ab,ti,kw OR (Carotid Artery Narrowings):ab,ti,kw OR (Narrowings, Carotid Artery):ab,ti,kw OR (Carotid Artery Stenosis):ab,ti,kw OR (Artery Stenoses, Carotid):ab,ti,kw OR (Artery Stenosis, Carotid):ab,ti,kw OR (Carotid Artery Stenoses):ab,ti,kw OR (Stenosis, Carotid Artery):ab,ti,kw OR (Internal Carotid Artery Stenosis):ab,ti,kw OR (External Carotid Artery Stenosis):ab,ti,kw OR (Stenosis, External Carotid Artery):ab,ti,kw OR (Carotid Artery Plaque):ab,ti,kw OR (Artery Plaque, Carotid):ab,ti,kw OR (Artery Plaques, Carotid):ab,ti,kw OR (Carotid Artery Plaques):ab,ti,kw OR (Plaque, Carotid Artery):ab,ti,kw OR (Plaques, Carotid Artery):ab,ti,kw OR (Carotid Ulcer):ab,ti,kw OR (Ulcer, Carotid):ab,ti,kw |
| **2** | (Medicine, Chinese Traditional):ab,ti,kw OR (Zhong Yi Xue):ab,ti,kw OR (Chung I Hsueh):ab,ti,kw OR (Hsueh, Chung I):ab,ti,kw OR (Chinese Medicine, Traditional):ab,ti,kw OR (Chinese Traditional Medicine):ab,ti,kw OR (Traditional Chinese Medicine):ab,ti,kw OR (Traditional Tongue Diagnosis):ab,ti,kw OR (Tongue Diagnoses, Traditional):ab,ti,kw |
| **3** | (randomized controlled trial):ab,ti,kw OR (randomized):ab,ti,kw OR (placebo):ab,ti,kw |
| **4** | #1 AND #2 AND #3 |

Searching Strategies (Embase)

| **Search number** | **Query** |
| --- | --- |
| **1** | 'Carotid Stenosis':ab,ti OR 'Carotid Stenoses':ab,ti OR 'Stenoses, Carotid':ab,ti OR 'Stenosis, Carotid':ab,ti OR 'Carotid Artery Narrowing':ab,ti OR 'Artery Narrowing, Carotid':ab,ti OR 'Artery Narrowings, Carotid':ab,ti OR 'Carotid Artery Narrowings':ab,ti OR 'Narrowings, Carotid Artery':ab,ti OR 'Carotid Artery Stenosis':ab,ti OR 'Artery Stenoses, Carotid':ab,ti OR 'Artery Stenosis, Carotid':ab,ti OR 'Carotid Artery Stenoses':ab,ti OR 'Stenosis, Carotid Artery':ab,ti OR 'Internal Carotid Artery Stenosis':ab,ti OR 'External Carotid Artery Stenosis':ab,ti OR 'Stenosis, External Carotid Artery':ab,ti OR 'Carotid Artery Plaque':ab,ti OR 'Artery Plaque, Carotid':ab,ti OR 'Artery Plaques, Carotid':ab,ti OR 'Carotid Artery Plaques':ab,ti OR 'Plaque, Carotid Artery':ab,ti OR 'Plaques, Carotid Artery':ab,ti OR 'Carotid Ulcer':ab,ti OR 'Ulcer, Carotid':ab,ti |
| **2** | 'Medicine, Chinese Traditional':ab,ti OR 'Zhong Yi Xue':ab,ti OR 'Chung I Hsueh':ab,ti OR 'Hsueh, Chung I':ab,ti OR 'Chinese Medicine, Traditional':ab,ti OR 'Chinese Traditional Medicine':ab,ti OR 'Traditional Chinese Medicine':ab,ti OR ' Traditional Tongue Diagnosis ':ab,ti OR 'Tongue Diagnoses, Traditional':ab,ti |
| **3** | 'randomized controlled trial':ab,ti OR 'randomized':ab,ti OR 'placebo':ab,ti |
| **4** | #1 AND #2 AND #3 |

Searching Strategies (Web of Science)

| **Search number** | **Query** |
| --- | --- |
| **1** | TI= (Carotid Stenosis OR Carotid Stenoses OR Stenoses, Carotid OR Stenosis, Carotid OR Carotid Artery Narrowing OR Artery Narrowing, Carotid OR Artery Narrowings, Carotid OR Carotid Artery Narrowings OR Narrowings, Carotid Artery OR Carotid Artery Stenosis OR Artery Stenoses, Carotid OR Artery Stenosis, Carotid OR Carotid Artery Stenoses OR Stenosis, Carotid Artery OR Internal Carotid Artery Stenosis OR External Carotid Artery Stenosis OR Stenosis, External Carotid Artery OR Carotid Artery Plaque OR Artery Plaque, Carotid OR Artery Plaques, Carotid OR Carotid Artery Plaques OR Plaque, Carotid Artery OR Plaques, Carotid Artery OR Carotid Ulcer OR Ulcer, Carotid) |
| **2** | TI= (Medicine, Chinese Traditional OR Zhong Yi Xue OR Chung I Hsueh OR Hsueh, Chung I OR Chinese Medicine, Traditional OR Chinese Traditional Medicine OR Traditional Chinese Medicine OR Traditional Tongue Diagnosis OR Tongue Diagnoses, Traditional) |
| **3** | TI= (randomized controlled trial OR randomized OR placebo) |
| **4** | #1 AND #2 AND #3 |
